# Spheroid Engineering in Microfluidic Devices

**DOI:** 10.1021/acsomega.2c06052

**Published:** 2023-01-18

**Authors:** Atakan Tevlek, Seren Kecili, Ozge S. Ozcelik, Haluk Kulah, H. Cumhur Tekin

**Affiliations:** † METU MEMS Research and Application Center, Ankara 06800, Turkey; ‡ The Department of Bioengineering, Izmir Institute of Technology, Urla, Izmir 35430, Turkey; § The Department of Electrical and Electronics Engineering, Middle East Technical University, Ankara 06800, Turkey

## Abstract

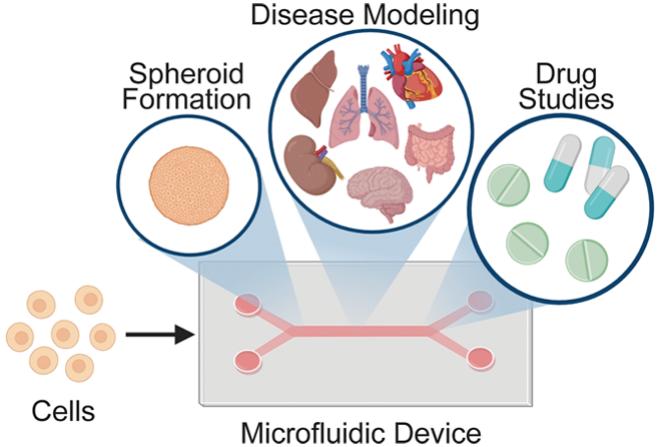

Two-dimensional (2D) cell culture techniques are commonly employed to investigate biophysical and biochemical cellular responses. However, these culture methods, having monolayer cells, lack cell–cell and cell–extracellular matrix interactions, mimicking the cell microenvironment and multicellular organization. Three-dimensional (3D) cell culture methods enable equal transportation of nutrients, gas, and growth factors among cells and their microenvironment. Therefore, 3D cultures show similar cell proliferation, apoptosis, and differentiation properties to *in vivo*. A spheroid is defined as self-assembled 3D cell aggregates, and it closely mimics a cell microenvironment *in vitro* thanks to cell–cell/matrix interactions, which enables its use in several important applications in medical and clinical research. To fabricate a spheroid, conventional methods such as liquid overlay, hanging drop, and so forth are available. However, these labor-intensive methods result in low-throughput fabrication and uncontrollable spheroid sizes. On the other hand, microfluidic methods enable inexpensive and rapid fabrication of spheroids with high precision. Furthermore, fabricated spheroids can also be cultured in microfluidic devices for controllable cell perfusion, simulation of fluid shear effects, and mimicking of the microenvironment-like *in vivo* conditions. This review focuses on recent microfluidic spheroid fabrication techniques and also organ-on-a-chip applications of spheroids, which are used in different disease modeling and drug development studies.

## 1 Introduction

Inside the body, cells have self-assembly organization capability via intercellular signaling to constitute 3D tissues hierarchically. The extracellular matrix (ECM) formed by the cells guarantees respectable properties such as cell viability, cell functionality, cell differentiation, and mechanical properties. Cell culture studies have been performed to achieve various diagnoses and treatment systems specific to the human body by imitating many physiological events that occur in the body under laboratory conditions.^1^ For this purpose, cells are grown on 2D plastic surfaces, namely, Petri dishes, well plates, or specific culture flasks, with the presence of a culture medium composed of various nutrients, ions, and salts at 37 °C. Also, the culture medium is generally supplemented with serums, antibiotics, and proteins or amino acids according to the requirement of the cell being cultured. Upon cells reaching confluency, they are collected from the cultured surface by enzymes such as trypsin and are obtained as suspended. This conventional cell culture technique has still been successfully used with slight modifications since its discovery in 1907.^2^ The cell culture technique is of great importance for the biomedical community, especially in tissue engineering and regenerative medicine, because it has been successfully used in preclinical research in the area pertaining to various vaccine and drug developments, cytotoxicity evaluations, biocompatibility assessments, and testing of the therapeutic effects of various molecules.^3^ The key benefits of 2D cultures are that they are quite applicable, inexpensive, and not very sensitive to the changes of the operator and their environmental conditions are easily controlled.^4,5^

Although 2D cell culture techniques have still been commonly used because of the above-mentioned advantages, several drawbacks cannot be overcome, such as deteriorated cell signaling due to loss of cell phenotype, delayed response to stimuli found in the external environment due to decreased cellular polarity, and nonhomogeneous distribution of the nutrients, metabolites, signal molecules, and various gases due to the inability to imitate the microenvironment.^6,7^ Because complex cellular signaling between cells and their matrix cannot be replicated in a 2D culture, *in vitro* experimental data obtained from 2D cultures cannot be fully represented in *in vivo* conditions.^8^

A 3D cell culture, which enables cells to expand and communicate in three dimensions with the surrounding extracellular milieu, has been suggested to fulfill unmet physiological needs and culture conditions in traditional cultures.^9^ Basically, an optimum 3D culture should support cell growth by simultaneously providing the requisite nutrients, moisture, and oxygen and removing the degradation products.^10^ 3D cultures have great advantages compared to 2D cultures in terms of cell morphology, cell differentiation, viability, cell proliferation, stimuli response, drug metabolism, gene and protein expression, cellular functionality, and *in vivo* relevance.^1,11^ For a 3D culture, both conventional methods and new technologies based on microfluidics can be applied. Conventional methods have limited control over the size and geometry of 3D cell spheroids.^12^ However, robust, reproducible, and high-throughput 3D cell spheroid formation can be achieved using microfluidic technologies.^13^ Spheroids generated using advanced 3D culture techniques have been emerging as tissue precursors used to develop a variety of on-a-chip tissue and disease models, particularly drug delivery systems, by simulation of the complex multicellular architecture, barriers to mass transport, extracellular matrix synthesis, various protein and gene expressions, and *in vivo* physiological conditions.^14^

Although spheroid formation and its applications have been discussed in the literature,^15−18^ only a few reviews examining microfluidics systems for spheroid research have been done.^19−23^ Here, conventional approaches employed in the formation of spheroids are first addressed to reveal the importance of microfluidic technology in spheroid engineering. Subsequently, existing microfluidic systems utilized in the fabrication of spheroids, as well as contemporary literature examples, are presented in detail by stressing each technique’s benefits and limitations. Moreover, not only the microfluidic techniques used in spheroid production but also the diversity of cell types used in spheroid production within microfluidic systems, the parameters to be considered in culture conditions, the use of biomaterials in spheroid production, and the issues that should be considered in the design of microfluidic systems are discussed. Finally, the organ-on-a-chip applications are critically reviewed as applications of microfluidic-based chip systems used in spheroid engineering for disease modeling and drug-screening studies.

## 2 Conventional 3D Cell Culture Techniques Used for Spheroid Engineering

**Figure 1:**
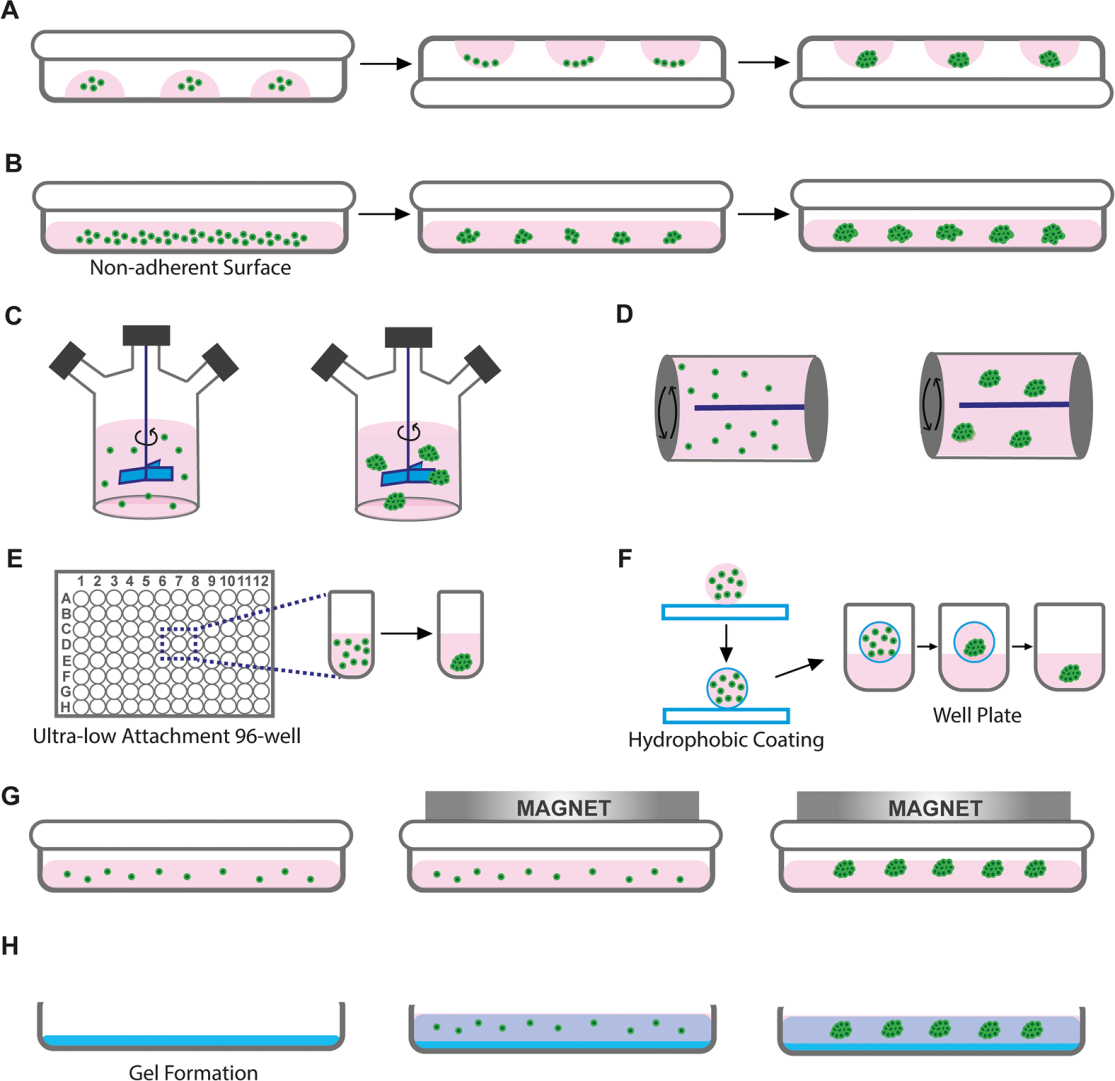
Conventional 3D cell culture techniques for spheroid formation: (A) hanging drop, (B) liquid overlay, (C) spinner bioreactors, (D) rotating bioreactors, (E) ultralow attachment plates, (F) liquid marbles, (G) magnetic levitation, and (H) gel embedding.

There are many techniques proposed for 3D cell cultures, such as liquid overlay, ultralow attachment plates, liquid marbles, hanging drop, spinner bioreactors, rotational bioreactors, magnetic levitation, and gel embedding.^24,25^ All these conventional techniques basically aim to provide cellular self-organization in 3D and are illustrated in Figure 1, comparatively.

In the hanging-drop method, small droplets of cell suspension prepared in a culture medium are formed on the lid of a Petri dish with the help of a micropipette, and the lid is closed on a Petri dish containing water to prevent the droplets from drying (Figure 1A). Inside the hanging droplet, the cells are subjected to gravity, and microtissues are formed with the self-assembly organization. The hanging-drop technique is relatively advantageous because it enables the acquisition of microtissues in relatively uniform sizes, it is practically applicable, and the environmental conditions can be easily controlled. However, this technique is inadequate for mass production and requires intensive labor.^26^

In the liquid overlay technique, the nonadhesive substrate-coated culture surfaces are practically used without the need for special equipment so that the cells aggregate to form microtissues instead of attaching them to the surface (Figure 1B). The main limitations of this technique are the size heterogeneity of the obtained microtissues, uncontrolled cell distribution and composition, and intensive labor requirements.^27,28^ In spinner bioreactors, cells in the culture medium are exposed to constant stirring, which provides shear stress that forms a 3D structure by hindering the cells from sticking to the bioreactor walls (Figure 1C). This technique is highly suitable for working with different cells at the same time to obtain heterotypic microtissue formation. It also provides scalable output, simple fabrication, and a long culture process.^29^ On the other hand, the prolonged shear forces that occurred from the spinning process may drive cells to apoptosis.^30^ Also, depending on the spinning speed, rupture or disintegration may arise in formed microtissues over time.^27,31^ Moreover, rotating bioreactors allow cellular self-organization to obtain microtissues^32^ (Figure 1D). Also, they provide long-term controllable culture conditions and allow the co-culture of various cell types.^33^ Although rotating bioreactors are very effective in the transport of nutrients, the uniform distribution of different gases, and the removal of waste via the perfusion method, formed cell aggregates have nonhomogeneous size distribution.^32,33^

One conventional approach for creating spheroids is the use of ultralow attachment plates (ULA), which is based on employing hydrophobic substrates to hinder cell adhesion and cause the cells to interact with each other to form spheroids^34−36^ (Figure 1E). The main limitation of this technique is the inhomogeneous size distribution of produced spheroids, which may lead to inconsistent results in experimental studies.^37^

The liquid marble (LM) technique has been developed for spheroid formation by using the surface wettability features of the material employed in a manner similar to that of the liquid overlay approach^38^ (Figure 1F). In contrast to the liquid overlay approach, the goal of LM is to cover a drop of liquid with hydrophobic powder particles to create a thin, porous, elastic hydrophobic outer shell. The coating material provides a closed spheroid formation that does not support cell adhesion and allows the cells trapped inside to freely interact with each other and to self-assemble into spheroids over time. Polytetrafluoroethylene powders are the most frequently used material in the literature as a hydrophobic coating material.^39^ Spheroids from different cells were successfully fabricated using LMs.^40^ However, the LM technique suffers from undesirably high evaporation and is difficult to handle, which can affect the integrity, homogeneity, and size of the spheroids.^41^

Magnetic levitation, one of the 3D cell culture techniques, is based on the principle of imitating the nongravity environment with magnetic forces^42^ (Figure 1G). The magnetic levitation technique may be used to form cell aggregates through positive magnetophoresis based on the directing cells labeled with magnetic beads or negative magnetophoresis based on the concept of controlling diamagnetic cells in a paramagnetic medium.^43,44^ The magnetic forces used in these systems are reported to provide uniformly sized and shaped cell aggregates.^43,45,46^ In addition, these microtissues, formed as a result of directed magnetic fields, can also be easily guided for tracking or imaging studies by virtue of the same magnetic forces.^47^

The gel-embedding technique is based on the encapsulation of the cells within a hydrogel defined as cross-linked polymer molecules (Figure 1H). With a wide variety of formulations, biophysical characteristics, and biological functions, hydrogels can be engineered and can thus substitute for several features of native ECMs.^48^ For the preparation of hydrogels, various types of polymers, from natural to synthetic, have been used. Although natural polymers are generally preferred because of their similarity to the ECM structure, biocompatible nature, and rich protein content, synthetic polymers have often been chosen because of their adjustable mechanical and degradable properties, easy production process, and nonimmunogenic nature.^49,50^ In addition, parameters such as viscoelastic property, structural integrity, stability, and degradation behavior of the developed gels should be taken into account for the formation of spheroids.^51,52^

Ultimately, the labor-intensive and time-consuming nature of these 3D conventional culture techniques, in addition to the difficulty of changing the culture medium, the low-yield production, the difficulty of controlling the spheroid size, and cross-contamination issues, have prompted researchers to investigate microfluidic approaches for spheroid production.^53,54^

## 3 Spheroid Engineering in Microfluidic Systems

**Figure 2:**
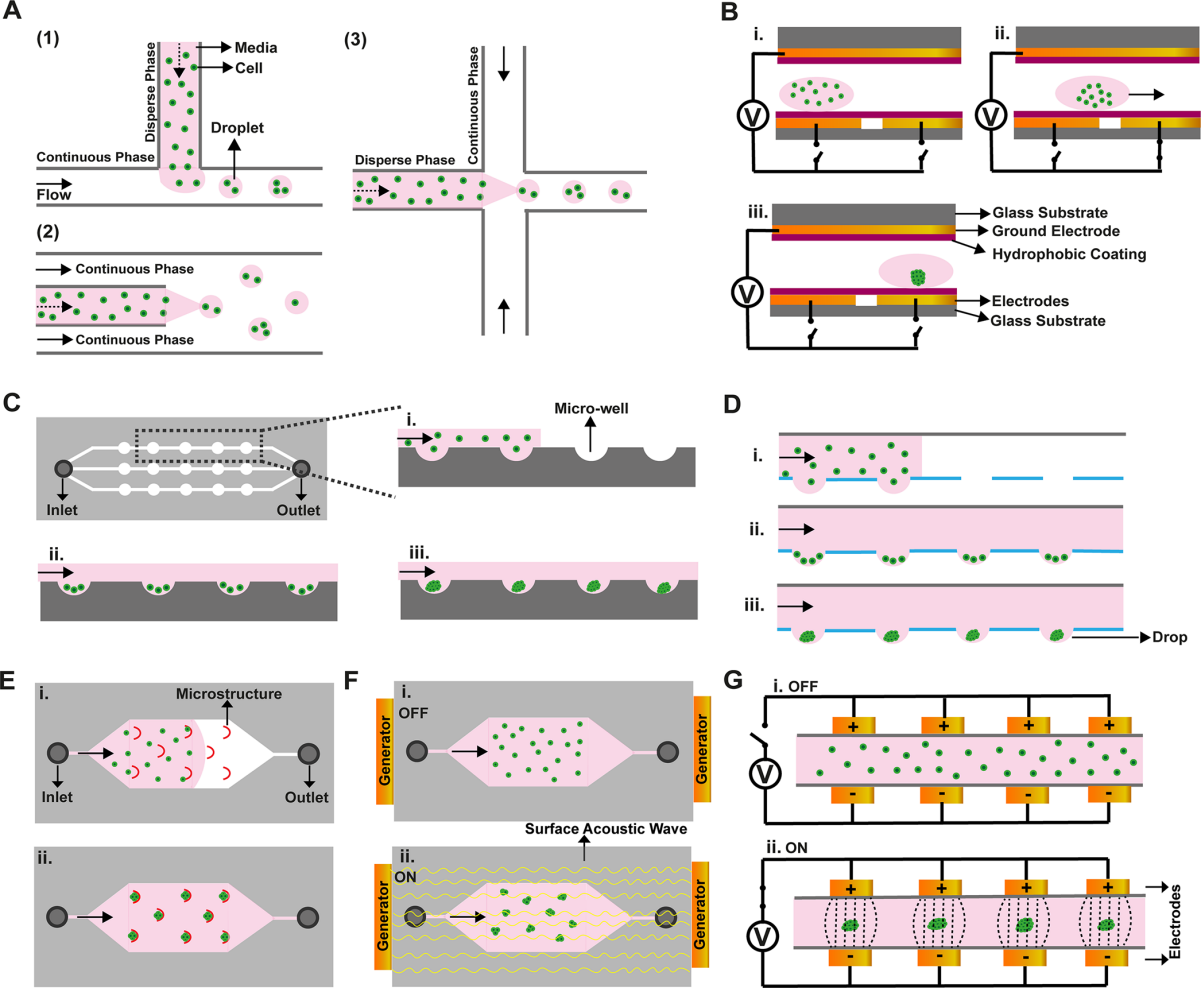
Illustrations for microfluidic-based methods in spheroid formation. (A) Droplet-based methods: (1) T-junction breaks up the dispersed phase with a sheath flow; (2) in co-flowing, the dispersed phase is generated with a needle or tube; and (3) flow focusing uses two sheath flows for breaking of the dispersed phase. (B) Electrowetting generated with an electrode pattern. (C) Microwells in channels. (D) Microfluidic hanging drop with open wells in the channel. (E) Microstructure for cell trapping. (F) Acoustic manipulations. (G) Dielectrophoresis with a nonuniform electrical field. (i), (ii), and (iii) represent spheroids formation steps in all images.

Spheroid formation has been applied in microfluidic systems to examine cell characteristics and microtissue formation and is used in different applications such as tissue engineering,^55^ regenerative medicine,^56^ drug screening,^57^ and disease modeling.^58^ These systems enable the use of several processes and manipulations that have typically been hard to handle with conventional methods. For instance, laminar and turbulent flow and microdroplet formation show great advantages in the formation of spheroids.^20,22^ Moreover, forming 3D tissue in a dynamic environment in a microfluidic chip offers high cell viability compared to that in static conditions.^59^ Microfluidic systems are also advantageous in supplying an adequate amount of nutrients and oxygen to cells in a well-controlled environment. Furthermore, with these systems, the size and composition of spheroids can be controlled precisely in a low-cost design using only a low amount of reagents.^60^ Hence, microfluidic-based tools could afford great benefits in spheroid engineering compared to conventional 3D cell culture methods. Here, we will focus on different microfluidic techniques used for spheroid generation (Figure 2 and Table 1).

**Table 1:** Summary of Spheroid Fabrication Techniques Used in Microfluidic Systems^a^

technique	cell type used	biomaterial type used	obtained spheroid size/volume/area	no. of fabricated spheroids/spheroid formation time	application area	reference
droplet	rat hepatoma continuous cell line (H4-II-EC3)	agarose	72.9 ± 18.6 μm	500 spheroids in 11 h	3D cell culture	(61)
	murine colorectal carcinoma cell line (CT26.WT)	alginate	up to 0.1 mm^3^	>1000 droplets/s	anticancer therapies	(62)
	human cervical carcinoma cells (HeLa)	Matrigel and alginate	138 ± 20 μm	NR/96 h	anticancer therapies	(63)
	human mesenchymal stem cells (hMSC)	alginate/arginine-glycine-aspartic acid (-RGD)	30–80 μm	NR/150 min	tissue engineering	(64)
	diffuse large B-cell lymphoma cell line (SUDHL-10); fibroblast cell line (HS-5); peripheral blood mononuclear cells (PBMCs)	alginate/PuraMatrix	350 ± 25 μm	250 spheroids/NR	drug screening	(65)
	embryo-derived teratocarcinoma cell line (P19)	alginate	111 μm	NR/48 h	tissue engineering	(66)
	human breast adenocarcinoma cell line (MCF-7); human mammary fibroblast cells (HMF)	alginate	NR	200 spheroids/min /7 days	drug screening	(67)
	primary human bone-marrow-derived mesenchymal stem cells (hBMSC)	polyethylene glycol-diacrylic (PEGDA)	<50 μm	NR/28 days	3D cell culture	(68)
	human glioblastoma cells (U87MG)/mouse neural stem cells (NE-4C)		100–130 μm	42,000 spheroids/1 h	regenerative therapy	(69)
	human glioblastoma cell line (U-251)	polyethylene glycol (PEG)/RGDs	118–480 μm	NR/1 h	drug screening	(70)
	hMSC	poly(vinyl alcohol) (PVA)	90 μm	NR/4 weeks	drug screening	(71)
	MCF-7/human fibroblast cell line (HS-5)	alginate	170 μm	1000 spheroids/48–72 h	anticancer therapies	(72)
	human embryonic kidney cells (HEK293)/human bladder cancer cell line (RT4)/human epidermoid carcinoma cells (A431)	PEG–perfluoropolyether (PEG–PFPE)	NR	85,000 spheroids/1 h	3D cell culture	(73)
electrowetting	Madin-Darby canine kidney epithelial cells (MDCK)	Geltrex/agarose/polyacrylamide/alginate/type I collagen	20 μm	NR/1 day	3D cell culture	(74)
	HepG2/mouse embryonic fibroblast cells (NIH-3T3)	type I collagen	NR	NR	drug screening	(75)
	mouse bone-marrow-derived mesenchymal stem cells (BM-mMSC)/ HT-29		up to 400 μm	NR/in 72 h	3D cell culture	(76)
microwell	HeLa/human umbilical vein endothelial cells (HUVECs)	PEG	50–300 μm	NR/up to 36 h	anticancer therapies	(77)
	human dermal fibroblasts (hDFs)	cellulose nanocrystals and gelatin	∼150 μm	2400 spheroids/5 day	drug screening	(78)
	MCF-7	agarose	200–600 μm	up to 175 spheroids/72 h	drug screening	(79)
	human lung carcinoma epithelial cell line (A549)/ human osteoblasts/patient-derived spine metastases cells (BML)		up to 250 μm	10 spheroids/5 day	personalized medicine	(80)
	human high-grade glioma cells (UVW)/human prostate cancer cell line (LNCaP)/patient biopsy-derived prostate cancer cells		50–150 μm	240 spheroids/48 h	personalized medicine	(81)
	rat embryonic fibroblast cells (REF52)/Madin-Darby canine kidney (MDCK) cells	fibronectin/collagen	40–100 μm	NR	tissue engineering	(82)
	human colorectal adenocarcinoma cell line (HT29)		up to 250 μm	25 spheroids/7 days	drug screening	(83)
	human colorectal carcinoma cell line (HCT116)/human glioma cell line (U87)		150–200 μm	50 spheroids/48 h	drug screening	(84)
	HT-29 cells		130–250 μm	20 spheroids/6 days	anticancer therapies	(85)
	MCF-7/U87	agarose	up to 500 μm	40 spheroids/5 days	anticancer therapies	(86)
	human hepatocellular carcinoma cells (HepG2-C3A)	gelatin methacryloyl (GelMA)	191 ± 10 μm	10,000 spheroids/5 days	drug screening	(87)
	human lung adenocarcinoma cells (A549)/human lung fibroblast cells (MRC-5)	type I collagen	NR	28 spheroids/72 h	regenerative therapy	(88)
	human articular cartilage cells (hACs)		NR	NR/14 days	tissue engineering	(13)
	murine ES cell (ES-D3)/human hepatocellular carcinoma cell (HepG2)/monkey kidney epithelial fibroblast (COS-7)		up to 210 μm	5000 spheroids/24 h	3D cell culture	(89)
	human glioma cell line (U87)	PEGDA	361.3 ± 36.2 μm	24 spheroids/24–48 h	drug screening	(90)
	human mesenchymal stem cells (hMSC)	chitosan/polydopamine	up to 500 μm	NR/5 days	3D cell culture	(91)
	human metastatic breast adenocarcinoma cell line (MDA-MB-231)/human nontumorigenic mammary epithelial cell line (MCF-10A)	type I collagen	100 μm	1296 spheroids/5–6 days	3D cell culture	(92)
	MCF-7/HCT-116		180 μm	240 spheroids/24 h	3D cell culture	(93)
	human colon adenocarcinoma cell line (Caco-2)/normal human dermal fibroblasts (NHDF)/human alveolar basal adenocarcinoma cell line (A549)/human hepatocellular carcinoma cell line (HepG2)		up to 828.7 ± 49.5 μm	360 spheroids/72 h	tissue engineering and drug screening	(94)
	human hepatoma cells (Huh-7)	Geltrex	160 μm	120 spheroids/24 h	drug screening	(95)
	A549/human fetal lung fibroblast cell line (MRC-5)	type I collagen	142.3 μm^2^	84 spheroids/24 h	tissue engineering	(96)
hanging drop	mouse embryonic stem cells (mESC)/human lung cancer cell line (A541)/human leukemia cell line (HL-CZ)		up to 250 μm	234 spheroids/24 h	3D cell culture	(97)
	mouse embryonic stem cells (mESCs) and human MDA-MB-231 and MCF-7 breast cancer cells	Matrigel	up to 730 ± 27 μm	16 spheroids/10 days	3D cell culture	(98)
	human epithelial carcinoma cell (A431.H9)		up to 0.16 μL	384 spheroids/7 days	drug screening	(99)
	mouse embryonic stem cells (mESCs; ES-D3)		80–120 μm	72 spheroids/24 h	tissue engineering	(100)
	human Wharton’s jelly-derived mesenchymal stem cells (WJ-MSC)		up to 500 μm	24 spheroids/7 h	3D cell culture	(101)
	human umbilical cord blood-derived MSCs/mouse podocyte cells		up to 500 μm	49 spheroids/24 h	tissue engineering	(102)
	human glioblastoma cell lines (LN229 and PDX)		4 nL	900 spheroids/1 h	drug screening	(103)
	human synovial sarcoma-derived cell line (HS-SY-II)/human umbilical cord-derived mesenchymal stem cells (UC-MSCs)		NR	NR/24 h	anticancer therapies	(104)
	human hepatocellular carcinoma cell line (MHCC97-H)		522 ± 40 μm	26 spheroids/3 days	3D cell culture	(105)
microstructures	HepG2/mouse fibroblast cells (Balb/c-3T3)	PEGDA	NR	56 spheroids/48 h	drug screening	(106)
	MCF-7/HepG2		up to 1 × 10^–2^ mm^3^	16 spheroids/2 days	drug screening	(107)
	human glioblastoma cell line (U87-MG)	gelatin-based electrospun nanofibers	220 μm	NR/2 days	anticancer therapies	(108)
	human breast tumor cells (LCC6/Her-2)	alginate	250 μm	NR/4 days	anticancer therapies	(109)
	human breast cancer cell lines (BT49 and T47D)	basement membrane extract (BME)	120 μm/125 μm	11 spheroids/1 day	drug screening	(110)
	HT29 human colon carcinoma		200–550 μm	∼10000 spheroids/10 day	drug screening	(111)
	human alveolar basal adenocarcinoma cell line (A549)		NR	16 spheroids/72 h	3D cell culture	(112)
	human breast cancer stem cells (BCSCs)	Matrigel	NR	NR/5 days	tissue engineering	(113)
acoustic	MCF-7/A549/human ovarian cancer cell line (A2780)/murine embryonic carcinoma cell line (P19)		NR	6000 spheroids/24 h	3D cell culture	(114)
	murine endothelial cell line (2H11)/ NIH 3T3/human embryonal kidney cell line (293FT)		185.2 ± 50 μm	NR/9 h	tissue engineering	(115)
dielectrophoresis	human T lymphocyte cells (Jurkat)/mouse stromal cells (AC3)		at least 100 μm	NR/5 min	3D cell culture	(116)
	human hepatoma cell line (HuH7)		50–100 μm	NR/45 min	3D cell culture	(117)

a NR: not reported.

### 3.1 Droplet-Based Methods

In biomedical studies, droplet-based microfluidic methods have been used for different applications where size-controllable monodisperse droplets can be generated and manipulated (Figure 2A).^19^ To create an identical spheroid, the fabrication of uniform-sized droplets is crucial. The spheroids are formed by encapsulating cells into the droplets, and the size of spheroids can be regulated by droplet size. The geometry of the microfluidic chips controls the droplet generation, and the rate of generation can be regulated by the type of fluid inside the microchannels.^61^ Encapsulating cells and forming spheroids in such microfluidic systems can be achieved by using different liquids to form single- or double-emulsion droplets.^62^ Single emulsions are the most basic type of emulsion, consisting of a liquid droplet dispersed in another fluid and frequently stabilized by surfactants. Water-in-oil systems, such as “cell suspension droplets in oil”, are well-known examples of single-emulsion techniques.^63^ Double emulsions are liquid dispersion systems, also known as emulsions of emulsions, in which droplets of one dispersed liquid (emulsion, microemulsion, liposome, etc.) are dispersed in another liquid (water or oil), resulting in double-layered liquid droplets.^118,119^ Water-in-oil-in-water systems, such as “cell suspension droplets-in-oil-in-water” are the most commonly applied double-emulsion techniques.^64^ The selection of the culture medium, physiological fluid, or hydrogel for the entrapment of cells and the determination of the oil phase is of crucial importance for generating 3D cell-laden spheroids.^19^ To accomplish spheroid formation in microfluidic systems, geometric models such as a T-junction, co-flowing, and flow focusing have been widely investigated.^22,61−73^

A T-junction is the most used geometry for its simple fabrication, operability, and controllability for creating monodisperse droplets. Principally, the T-junction arrangement enables the formation of droplets by the shear stress produced by two immiscible flowing fluids at the intersection of two consecutive microchannels.^120^ Here, two immiscible liquids are defined as the continuous phase and the other as the dispersed phase. The intersection of two distinct microchannels carrying the continuous and dispersed phases can be positioned between 0° and 90°.^121^ The continuous phase is usually composed of oils or organic solvents that are more viscous than water and immiscible with water, whereas the dispersed phase is usually water or water-based physiological fluids.^122^ T-junction methods have previously been used in forming identical droplets for both cells and drug encapsulations in microfluidic platforms for drug-screening applications.^57^ In a previous study, the combination of an alginate and PuraMatrix hydrogel system was used in a droplet-integrated microfluidic chip to entrap the cancer cells, fibroblasts, and lymphocytes at the same time to achieve tumor spheroids for immunotherapy studies.^65^ Lenalidomide anticancer drug was tested on the obtained tumor spheroids at a size of 350 ± 25 μm. On the basis of the findings, the microfluidic system allows not only spheroid production but also measurement of cell proliferation, cell–cell interaction, and cytotoxic effects of anticancer drugs on formed spheroids semiautomatically.

The co-flowing approach allows the formation of micrometer-sized droplets by interbedding the dispersed phase and continuous phase together.^123^ The phase difference between the continuous and dispersed phases causes the continuous phase to surround the dispersed phase, resulting in the formation of droplets.^124^ A microfluidic platform possessing a hillock structure based on the co-flowing technique was designed to investigate the spheroid formation in alginate microcapsules.^66^ The developed system was also used for the observation of the differentiation of P19 mouse embryonic carcinoma cells. It was reported that spheroids could be obtained in as little as 2 days in massive and uniform microcapsules with an average diameter of 111 μm.

In the flow-focusing technique, droplets are generated by orthogonally positioned channels that enable the droplets to flow in the direction of the dispersed phase channel.^125^ Briefly, unlike the co-flowing method, two vertical channels join the main flow channel at the same point in this technique, and droplet formation occurs at this cross-junction.^126^ In a study, size-controllable alginate-based spheroids were reported to possess MCF-7 tumor cells in the core and human mammary fibroblast cells (HMF) in the shell phase.^67^ The efficacies of two different anticancer drugs (Paclitaxel and Curcumin) on these produced spheroids were evaluated. The microfluidic-based fabricated spheroids exhibited higher drug resistance than the monolayer cell culture models but displayed equal resistance to those spheroids prepared using conventional methods.

### 3.2 Electrowetting Approaches

To construct discrete droplets, electrowetting approaches are used with microelectrode arrays (Figure 2B).^127^ The formation, mixing, and transportation of droplets are simple fluidic processes in digital microfluidics (DMF) using the electrowetting (EW) phenomenon without the use of pumping systems.^128^ Because of their easy automation/integration, rapid analysis, reduced sample volume, and addressability, DMF methods are advantageous when compared to conventional methods.^129^ Although digital microfluidics and electrowetting have not been widely used for spheroid manufacturing in recent years, particularly in terms of mass production, a few literature examples have been considered remarkable as a proposed approach for spheroid fabrication. For instance, DMF was used to form microgel on-demand arrays for constructing and culturing mesoscale cell spheroids.^74^ Madin-Darby canine kidney (MDCK) cells were seeded in microgels composed of Geltrex, type I collagen, and agarose, and these microgels were separately located in the hydrophilic site of the DMF device and were cultured for 4 days for 3D cell aggregation. In another study, the DMF system was developed to form organoids for drug-screening applications.^75^ Collagen scaffolds with a co-culture of HepG2 and NIH-3T3 cells were electrodynamically injected into the presented device, and organoids were formed. Afterward, acetaminophen (APAP) in different concentrations was introduced into the DMF platform, in which organoids were observed in terms of apoptosis and necrosis. According to the results, 10 mM APAP showed an apoptotic response, and both apoptotic and necrotic responses were observed for 20 mM APAP. In another study, an automated hanging-drop method with DMF was also presented for culturing bone-marrow-based mouse mesenchymal stem cell spheroids.^76^ For cell manipulation, this device contained top and bottom plates having electrodes and ground electrodes, respectively. The hanging drop formation was generated on the bottom plate with holes where cells tend to aggregate. With use of this method, spheroids with high viability and uniform size (up to 400 μm) were generated.

### 3.3 Microwell-Based Method

Microwell-based methods have generally been preferred because of their simple and easy-to-use process.^77^ With use of microfabrication processes such as photolithography, soft lithography, and etching, it is possible to create microwell arrays from different materials such as polyethylene glycol (PEG) and polydimethylsiloxane (PDMS) (Figure 2C).^130^ Because the cells do not attach to the surface in the microwells made in the appropriate dimensions, spontaneous spheroid formation occurs with sedimentation and accumulation of cells in these wells.^131^ In microwell-based approaches, both static and dynamic conditions, the deterioration of the structural integrity or cell loss may occur in developed spheroids during cell seeding, culture medium replacement, or various washing steps. However, the modification of the microfluidic channel dimensions (increased width or decreased height), revision of the reservoir geometry, changing of the microwell material, and use of integrated pumps may overcome these problems.^79,132−134^ Collection of the formed spheroids from the microwells is essentially required for postprocessing analysis such as biochemical, differentiation, or flow cytometry analysis. In this context, a suitable flow rate can be chosen to apply an adequate lift force for spheroid collection.^135^ Numerous studies have been conducted utilizing microwell-based approaches to generate uniformly sized spheroid structures in many fields, including 3D culture, drug screening, and tissue engineering.^13,77,79,81−91,93−96^ For instance, the construction of colorectal cancer cell (HT29) spheroids that have approximately 250 μm diameter at the end of the 7-day culture period was reported in a reversible microfluidic platform.^85^ Top and bottom parts were held together via cubic-shaped magnets that faced each other with opposite magnetic polarity. Alternatively, another microfluidic platform has reported the formation of multicellular spheroids having 50–100 μm diameter from biopsy-derived patient cells for drug-screening studies.^81^ This platform offers a self-perfusion ability that makes the platform an equipment-free device for the cultivation of formed spheroids. Furthermore, the concave microwells were used to form colon cancer (HCT116) cell spheroids having 120 μm diameter for the application of Irinotecan anticancer drug.^84^ It was reported that the cell viability, the spheroid number, and the spheroid uniformity were altered depending on the concentration level of the drug. Recently, tumor spheroids were formed using a human colon cancer cell line (HT29) in microwells under a continuous flow of culture medium.^83^ The developed platform allowed spheroid formations up to 250 μm diameter in size, and the cell viability was drastically decreased in spheroids throughout the 5-day culture in the presence of 5-fluorouracil anticancer drug. In another study, self-filling agarose-based microwells were demonstrated using inclined channels for analyzing drug toxicity in spheroids formed with MCF-7 breast cancer cells and also U87 brain tumor cells.^86^ It was shown that the developed tumor spheroids were found more resistant to the cytotoxic effect of the doxorubicin anticancer drug than the monolayer culture of the same cells. In another study, dense dermal fibroblast spheroids were created in biomimetic cellulose-nanocrystals-doped gelatin hydrogel under physiological flow conditions and to screen for alimunium (Al) in skin care products.^78^ It has been reported that 2400 spheroids with a diameter of 150 μm can be produced within 5 days in the proposed microfluidic platform. A microwell-based microfluidic biochip was also designed to generate uniform multicellular spheroids to study chemotherapeutic drugs.^80^ Ten spheroids with a size of 250 μm can be formed in 5 days on this chip and formed spheroids exhibit high resistance to anticancer drugs.

### 3.4 Hanging Drop

The hanging-drop method is known to be one of the widely used conventional methods for 3D spheroid formation in various applications.^97,100−105^ Becaue this easy-to-use method allowed the self-assembly of spheroids with the force of gravity and desired microenvironment for spheroids, the hanging-drop method has also been adapted to microfluidic systems (Figure 2D).^136^ For example, a PDMS-based microfluidic hanging-drop chip was reported to provide an automated long-term and high-throughput 3D cell culture for various applications such as cell differentiation, tissue engineering, developmental biology, and drug screening.^97^ Moreover, spheroids composed of Wharton’s jelly mesenchymal stromal cells (WJ-MSCs) were formed in a continuously perfused microfluidic hanging-drop platform.^101^ In this platform, the production of spheroids with a diameter of 500 μm was realized in 7 days; this could not be produced with the traditional hanging-drop approach because of the limited exchange of the culture medium. Furthermore, the conventional hanging-drop technique limits the formation of embryoid bodies because of the intensive workload and difficulty in changing the culture medium manually.^137^ To overcome these limitations, a PDMS-based microfluidic hanging-drop device containing microfluidic channels and wells was developed.^100^ A mouse embryonic stem cell suspension containing 3 × 10^5^ cells/mL was introduced in the channels under hydrostatic pressure. The cells were trapped in the wells of the microfluidic chip, and the 80–120 μm diameter of the embryoid body was easily formed in a 1-day cultivation. In another study, a pump-integrated PDMS microfluidic chip was designed to improve the flow control in the hanging-drop system.^136^ The physiological pulsative flow was achieved using the pneumatically actuated pump. Human iPS cell-derived cardiac microtissue spheroids were developed using this system, and formed spheroids exhibited a beating at a rate of 60–90 bpm, which is similar to the natural frequency of the human heart. A microfluidic hanging-drop-based spheroid co-culture system was also used to facilitate the formation and co-culture of embryoid bodies and tumor spheroids.^98^ In this study, spheroid generation up to 730 ± 27 μm was reported in Matrigel, and 16 spheroids per chip were produced in 10 days.^98^

### 3.5 Microstructures

The basic operation of this approach is based on delivering the cell suspension to the chip via a microfluidic channel and accumulating the cells in the microstructures patterned on the channel surface (Figure 2E).^138^ The accumulated cells form spheroids, and the medium can be constantly fed into the chip via microchannels.^139^ The diameters of the spheroids may be fine-tuned with the sizes and geometry of the microstructures. The major benefits of microstructures are that they protect cells from shear stress injury and allow delivery of nutrients to cells without damaging the spheroid structure.^106^ In microstructure-based systems, the pressure generated by the flow rate in front of the miniature structures within the chip is critical, and this stagnation pressure also generates a stagnation zone within the microstructures. Thus, although the culture media flow at a constant rate from the outside region of the structures, the static environment created within the structures promotes cell fusion and the formation of spheroids.^140^ Although there are not as many literature examples as the hanging-drop method and multiwell-based approach, there are promising examples using microstructures, which can be practically applied using photolithographic and molding approaches.^106−108,113,141^ For example, LCC6 breast cancer cells encapsulated with alginate beads having a size of approximately 250 μm were trapped via a U-shaped microstructure for spheroid formation. The effect of doxorubicin on the produced spheroids was also studied on the same chip.^109^ Furthermore, an *in vitro* breast tumor model containing an endothelial monolayer and ECM was reported on a chip to imitate a microvessel wall. The developed platform allowed the formation of uniformly sized multicellular tumor spheroids, and the platform was proposed further for use in drug-screening studies. After 14 days of culture, the average diameter of both BT549 and T47D breast cancer cell spheroids was determined to be around 180 μm, and formed spheroids showed good cell viability (>90%).^110^ In another study, a Si-based microfluidic chip with pyramid-like microstructures was used to fabricate and culture spheroids.^107^ With this device, 100 μm diameter MCF-7 breast cancer spheroids were produced in 2 days of culture, and spheroids’ sizes could be adjusted using different cell concentrations. A microfluidic device with U-shaped arrays was also utilized for a 3D cell culture of A549 cells under a continuous flow of culture medium.^112^ With this device, 16 spheroids can be produced in 72 h.

### 3.6 External Forces

The formation of spheroids can also be established with external forces such as acoustic actuation^142^ and dielectrophoresis (DEP).^143^ The major hurdle in dealing with these systems is the possibility of the cell damage caused by mechanical stresses inside the system. Hence, it is difficult to establish long-lasting cell spheroids using these approaches.^23^

#### 3.6.1 Acoustic Actuation

Acoustic-wave-based patterning can control the spatial position of cells. In this method, acoustic vibrations create a pressure gradient in a liquid, enabling simple, quick, noncontact, and precise 3D design of suspended cells in media or ECM-based hydrogels.^144^ With use of acoustic node assembly, many different complex cellular patterns, as well as spheroids, and also their patterns, can be fabricated rapidly.^144,145^ Low-frequency acoustic fields used in these systems prevent the heating of the solution and damage cells.

Acoustic waves allow label-free and contactless cell actuation in different fluidics. When compared to conventional spheroid formation methods, acoustic actuation (Figure 2F) allows higher throughput, easier size control, higher reproductivity/cell viability, and adaptability to different cell lines.^146^ For example, a PDMS-based microfluidic platform containing 60 parallel microfluidic channels with dimensions of 150 μm height and width and a depth of 3 mm was integrated with a surface sound acoustic wave (SSAW) generator.^114^ With this scheme, over 12,000 multicellular tumor spheroids can be generated in a few minutes. In another work, an acoustic-fluid device was used for the fabrication of homotypic and heterotypic spheroids without using any scaffold materials.^115^ In the device, the acoustic radiation force acted on the suspended cells to collect them in pressure nodes, and the spheroid size and cell components could be easily adjusted by changing the initial cell concentration and ratio. The device allowed spheroids to form a diameter of 185.2 ± 50 μm in 9 h.

#### 3.6.2 Dielectrophoresis

DEP in microfluidic platforms is a favorable method because of its fast, controllable, and efficient cell-patterning abilities.^134^ DEP is an electrical force categorized as negative and positive DEP that can be used for spheroid formation (Figure 2G).^147^ For instance, cell clusters were formed between the interdigitated electrodes using positive DEP.^116^ The aggregates of Jurkat cells and AC3 mouse stromal cells were generated by using a potential difference of 20 V at 1 MHz frequency. The developed method was reported to be suitable for investigating the cellular interactions in 3D cell aggregation in different sizes by adjusting the magnitude of the electrical field applied between electrodes. In another study, a negative DEP device with 3D upper and lower interdigitated electrodes was used to form HuH7 human hepatoma cell aggregates within 45 min.^117^

## 4 Organ-on-a-Chip Applications Using Spheroids

The integration of microfluidics and tissue engineering constitutes organ-on-a-chip (OOC) technologies.^148^ OOC technologies enable biological systems to be created in a controllable manner under certain physiological conditions. Consequently, the imitation of *in vivo* microenvironment conditions can be achieved so that tissue and organ physiology may be generated under *in vitro* conditions.^149^ Because of close contacts between cells and multicellular features, spheroids mimic paracellular signaling and physiological interface in heterogenic tissues or organs. Therefore, spheroids combined with OOCs can be a good candidate for accurate disease modeling.^150^ Furthermore, in preclinical pharmacological studies, spheroids employed in OOC systems may show more realistic drug responses compared to conventional cell culture methods by elucidating *in vivo* physiological conditions.^151^ Evolving OOC technology with spheroids provides researchers with several perspectives, especially in disease-modeling and drug-screening studies.

### 4.1 Disease Modeling

A disease model can be applied to animals or cells to exhibit all or a subset of both pathological processes and physiological functions found in corresponding human or animal illnesses. Examining disease models enables to gain a better understanding of how diseases arise and to evaluate new treatment methods.^152^ An important advantage of disease models is that the accuracy of epidemiological assumptions can be validated with the mathematical description of biological processes, allowing us to gain deeper insight into the onset and course of diseases.^153^ OOC systems could be utilized for disease modeling that could speed up the development of next-generation drugs and treatments for different diseases.^154^

Alzheimer’s disease is an age-related neurodegenerative disease that occurs because of amyloid-β accumulation.^155^ The 2D cell culture studies conducted in this field remain incapable of understanding the pathogenesis, progression, and underlying reasons for Alzheimer’s disease.^156^ In a study, a 3D brain model on a chip providing brain-like interstitial flow was developed to imitate a brain model with and without Alzheimer’s disease.^157^ The neurospheroids obtained from neural progenitor cells were cultured in the presence and absence of amyloid-β on a single chip. In the end, low cell viability, high neural disorders, and dysfunction were reported in neurospheroids exposed to amyloid-β. Hence, the investigation of pathophysiological properties of Alzheimer’s *in vitro* was enabled with the 3D brain-on-a-chip.

Nonalcoholic fatty liver disease (NAFLD) is one of the most common chronic illnesses, caused by aberrant fat accumulation, decreased protein production, and deficiencies in numerous biological functions.^158^ To investigate NAFLD pathogenesis, multicellular aggregates were prepared by using HepG2 and HUVECs.^159^ The function of the hepatocytes was evaluated in a chip in terms of albumin secretion and reactive oxygen species level following the multicellular aggregates exposed to free fatty acids. On the basis of the obtained results, the implementation of a diet without fat and an antisteatotic drug can enable regression of NAFLD.

The communication between the liver and the pancreas is another essential mechanism for the human body to control insulin-glucose regulation.^160^ OOC has great potential for investigating type-II diabetes mellitus *in vitro*. For instance, insulin secreted by islets positively affected glucose uptake from liver spheroids in the OOC system, and the glucose value in the medium was decreased.^161^

Ductal carcinoma *in situ* (DCIS) is a cancerous lesion formed by the collection of neuroplastic epithelial cells in the mammary duct.^162^ To model an early stage of breast cancer with DCIS, human breast cancer-on-a-chip was developed.^163^ For this purpose, breast cancer cell spheroids produced by a hanging-drop plate were collected and co-cultured with mammary ductal epithelial cells and fibroblasts to mimic the 3D structural organization of the human mammary duct. It was noted that the progression of malignancy of DCIS was successfully simulated via a compartmentalized 3D microfluidic device.

Inflammation and a variety of other disorders damage the integrity of endothelium and epithelial cellular barriers, which are critical for the selective transit of solutes and other molecules throughout the body.^164^ A co-culture of 3D tumor spheroids and cellular barriers was demonstrated in a microfluidic chip containing multiple wells with HT29 spheroids and an MDCK cellular barrier.^165^ The electrodes integrated into the chip enabled also transepithelial/transendothelial electrical resistance measurements to rapidly assess barrier sealing in real time.

### 4.2 Drug-Screening Studies

Typically, the drug development process is separated into three distinct phases: discovery, preclinical development, and clinical testing. The preclinical study is based on drug-screening research.^166^ Drug screening is the process of identifying and optimizing prospective medicines before the selection of a candidate drug for clinical trials.^109^ 3D cultures, particularly spheroids, have several benefits that have made them a popular tool for *in vitro* drug testing.^167^

For on-chip breast cancer drug delivery experiments, multicellular tumor spheroids (MCTSs) mimicking an ECM structure and monolayer endothelial cells mimicking a capillary channel structure were used.^110^ MCTSs were created by using BT549 (triple-negative breast cancer) and T47D (nontriple negative breast cancer) cells. Doxorubicin (DOX) was used as an anticancer drug with carbon-dots-based nanocarriers utilized for drug delivery. Transportation of the drug between the vessel and the ECM and its penetration into the MCTSs were tracked in real time using breast cancer-on-a-chip. In addition, *in situ* cytotoxicity testing was also performed in a single chip, and it was observed that controlled distributed DOX caused higher cytotoxicity in BT549 spheroids than in T47D spheroids. Furthermore, the efficiency of different chemotherapy drugs was also tested on breast cancer spheroids in a microfluidic chip.^168,169^ An evaluation of the efficacy of drugs cell viability analysis was conducted on bright-field micrographs of spheroids without using any staining process.

In a study based on vascularized cancer-on-a-chip, how the vascular structure of the tumor affected the drug resistance in tumor spheroids was investigated.^170^ For this purpose, the obtained multicellular tumor spheroids composed of human lung fibroblasts (hLFs), MCF-7, and HUVECs with and without vasculature were exposed to paclitaxel (PTX)anticancer drug. The results indicated that whereas PTX inhibited cell growth in static culture, it did not have the same impact in dynamic culture because of perfusion-induced vascularization.

In another study, tumor-on-a-chip was developed for the investigation of the effects of the PTX-loaded liposomes on an ovarian cancer model.^171^ The human ovarian cancer cell (SKOV3) spheroids formation, cultivation, and drug administration studies were conducted on the same chip. The four different formulations of liposomes were applied to the cells in 2D monolayer and 3D spheroids in different sizes under certain flow conditions. Consequently, small-sized tumor spheroids show better treatment efficacy with a low flow rate.

Temozolomide (TMZ) and bevacizumab (BEV) as clinical anticancer drugs were also tested for a high-grade and aggressive brain cancer model on a brain cancer chip containing glioblastoma multiform (GBM) spheroids.^172^ The drugs were applied to the primary human-derived GBM tumor spheroids both alone and in combination. According to the results, the combination of TMZ and BEV exhibited high treatment efficiency compared to that of a single TMZ application. This study has shown that the efficient combination of chemotherapy drugs, namely, drug cocktails, can be rapidly determined using a brain cancer-on-a-chip-based system.

To inspect a topically used antifungal drug terbinafine, reconstructed human skin and liver spheroids were cultured separately using a two-compartment TissUse’ HUMIMIC Chip2.^173^ The combination of skin and liver models enabled the measurement of toxic or metabolic reactions to the accumulated drug in the liver via transit through the skin barrier, which could not be assessed using conventional skin equivalents. In skin models, systemic terbinafine exposure boosted EGFR expression, whereas in the liver model on the same chip, it promoted apoptosis and decreased hepatic albumin expression.

## 5 Discussion

### 5.1 Cells and Biomaterials Used for Spheroid Engineering

To obtain 3D spheroids in a microfluidic system, various types of cells have been used (Table 1). In addition, co-culture studies have also been performed by using at least two cells at once to produce multicellular spheroids.^174^ There are several criteria to be considered in the evaluation of culture conditions according to used cell types in spheroid formation.^175^ For a spheroid generation, the optimal cell number, composition of the culture medium, and culture period should be determined. For co-culture experiments, the ideal cell ratio should also be identified. Additionally, the cellular process, such as doubling time, differentiation, metabolism, and survival rate, needs to be considered carefully to form a holistic microtissue.^176^ The success of the spheroid formation due to the cell types may differ.^177^ For example, osteosarcoma cells, human umbilical vein endothelial cells (HUVECs), human glioblastoma cells, tumor epithelial cells (TEC), and mesenchymal stem cells have been shown to proliferate more rapidly in 3D cultures than breast cancer cells, sheep-derived bone marrow stem cells, rat interior tibialis muscle cells, and smooth muscle cells.^1^ The size of formed spheroids could differ in the molecular characteristics of spheroids. For instance, HepG2 spheroids with a diameter of 200 μm showed higher albumin secretion compared to spheroids with a diameter of ≥300 μm.^178^ Hence, the size of spheroids should be precisely determined to get desired cellular functionality.

Microfluidic fabrication provides different options for fabricating large or small spheroids depending on applications.^14,179−181^ The type and density of the cells have been identified as the key issues concerning the diameter of the spheroids.^20^ For example, 10^7^ embryonic stem cells (ES)/mL, 10^7^ HepG2 cells/mL, and 10^5^ monkey kidney epithelial fibroblast (COS-7) cells/mL were fed into the microfluidic chip to generate spheroids.^182^ Although ES and COS-7 cells formed uniform aggregates at 16 and 24 h, HepG2 cells exhibited irregular spheroid formation at 24 h. The average diameter of the spheroids formed by using ES and COS-7 was measured as 80 μm, whereas the spheroids of HepG2 were 200 μm in diameter in a 3-day culture. Hence, the same initial cell density can result in the development of spheroids of varying diameters depending on the cell type. On the other hand, the cell density may cause different spheroid shapes depending on the type of the cell. For example, although MG63 and HepG2 cells were loaded into the chip system with 8 × 10^6^ cells/mL concentrations, MG63 cells showed a spherical shape, whereas HepG2 cells showed a nonspherical structure in a 5-day culture. Moreover, high cell density has the potential to clog microfluidic channels and result in the formation of nonspherical aggregates.^183^

Several matrixes have been used in spheroid-engineering research because of their biomimicry properties.^184^ Although various matrixes are produced and used in various shapes and sizes for 3D culture, the matrixes to be used within the framework of spheroid engineering in microfluidic devices are favored in a hydrogel configuration because of their formable properties.^185^ Natural polymers, synthetic polymers, and a variety of decellularized matrixes are the primary components utilized to create the hydrogel structure in this context.^186^ Natural polymers are of great interest in terms of hydrogel preparation because of their excellent biocompatibility, biodegradability, nontoxic natures, and resemblance to the form of native ECM.^187^ However, being physically weak and having components activating the immune or inflammatory response can discredit these materials.^187^ Thanks to their adjustable mechanical properties, biodegradation, and cross-linking density, the usage of synthetic polymers is more desirable than natural polymers.^188^ However, considerable attention should be paid to the problem of toxicity and biocompatibility that may arise from the reagents used in the synthesis phase.^189^ Apart from the polymers, decellularized-matrixes-derived hydrogels have been proposed for 3D culture studies on the basis of their site-specific biochemical and mechanical cues, the capability of regulating cellular behavior (e.g., attachment, proliferation, migration, and differentiation), and excellent resemblance to native tissue ECM.^190^ Nevertheless, several issues, such as loss of mechanical properties, pathogen transmission risk, deteriorated ECM structure or functionality, and immunogenic problems, need to be overcome.^191^ Because of these reasons, these materials have been utilized in combination rather than alone (Table 1). Hence, bringing these materials together can compensate and strengthen the properties of each material.

### 5.2 Design Parameters of Microfluidic Systems for Spheroid Engineering

The major goal of employing microfluidics in spheroid engineering is to transport cells to a predefined position on a chip while maintaining a continuous flow of culture media within the chip as the spheroid forms and maintains.^192^ The developed microfabrication techniques enable fabrication of complex and physiologically suitable microstructures in microfluidic chips that can affect the stability of the spheroid formation and size.^193^ For example, MPM H2052 cells were cultured in microchannels, including round-bottom and flat-bottom microwells.^194^ Because of the influence of the shape of the microstructure, round-bottom microwells have been reported to provide higher spheroid-forming efficiency than flat-bottom microwells. On the other hand, the symmetrical treelike structure employed in the design guaranteed that the hanging cells were retained homogeneously in eight microwells with the effect of gravity, resulting in the homogeneous size distribution of the spheroids created.

The advantages and limitations of spheroid fabrication techniques used in the microfluidic devices are summarized in Table 2. Microwell and droplet-based spheroid fabrication methods are widely used in microfluidic systems (Table 1). The main reason for this may be that droplet-based systems can produce thousands of spheroids in minutes or even seconds. Moreover, microwell-based systems do not require sophisticated lithographic manufacturing procedures, so they are cost-effective and practical systems. Additionally, microwell-based systems could be used to fabricate spheroids in different size ranges.^195^ Despite the growing interest in microfluidic hanging-drop and microstructure approaches for spheroid manufacturing, there are significant restrictions that preclude their utilization. For example, it is extremely difficult to establish a stagnation zone on the chip’s microstructures that do not harm cells.^196^ For this purpose, flow rate and shear stress in the chip must be controlled precisely to form and maintain the spheroids.^196^ Additionally, the recovery of spheroids produced by these technologies can be quite challenging.^20^ In the microfluidic hanging-drop technique, the nonuniform distribution of cells in each droplet can result in size and shape variations in formed spheroids.^101^ Additionally, one of the most fundamental issues with the microfluidic hanging-drop approach is that the culture medium cannot be replaced dynamically not to deteriorate the droplet structure.^101^ Electrowetting, acoustic, and dielectrophoresis methods are the least used techniques for spheroid fabrication. Electrowetting is limited in use because of challenges in designing and fabricating these electrowetting platforms.^125^ In spite of its advantages, such as flexible liquid handling and label-free manipulation, the acoustic method has downsides like contamination difficulties at the liquid–liquid interface and undesired heating issues.^127^ Dielectrophoresis also has tremendous potential for quick and precise cell manipulation.^197,198^ Nonetheless, with this approach, particular attention should be paid to the inability to establish cellular interactions due to the high electrical conductivity of the culture medium, as well as the risk of cell injury due to the strong electrical field.^199,200^ Although magnetic-based techniques were not utilized for spheroid fabrication in microfluidic channels, they have the potential to be used for fabricating self-assembled spheroids in a label-free manner.^42,201^

**Table 2:** Comparison of the Microfluidic-Based Methods for Spheroid Fabrication

methods	advantages	limitations
droplet-based	• create identical templates for spheroid formation	• resulting empty droplets (no cell containment)
	• single, double, and triple encapsulation variations	• insufficient nutrient supply
electrowetting	• easy automation and integration	• hard to design and fabricate these platforms
	• rapid analysis	
	• pump and valve-free operation	
microwell	• simple to operate	• cell loss and spheroid disruption during spheroid collection
	• controllable spheroid size	
microfluidic hanging drop	• self-assembly due to gravity	• high flow rate used to collect formed spheroids can damage spheroids
	• no cell adhesion observed on the surfaces	• nonhomogeneous number of cells in each hanging drop
microstructures	• reversible process enabling formation and collection of spheroids	• applying high flow rate can affect the spheroid formation time and make cells escape
	• efficient cell trapping due to high cellular interaction	
acoustic	• rapid spheroid formation enabling high cell viability	• possible cell damage due to heating problems while using high-frequency acoustic fields
	• simple and versatile technology to fabricate complex spheroids patterns in mild conditions	• complex fabrication processes while integrating acoustic wave generators on chip level
dielectrophoresis	• fast cell manipulation	• possible cell damage due to high electrical field
	• stable cell positioning	• high conductivity of culture medium may result in low cellular interactions and induce cell damage

Researchers have highlighted the necessity of a “well-controlled environment” and “more *in vivo* like circumstances” as advantages of microfluidic-based approaches over conventional procedures. However, when the studies presented in this review article are evaluated in this context, it is evident that the vast majority of them have focused on producing high-quality and large numbers of spheroids rapidly rather than providing a well-controlled microenvironment for the long-term culture of spheroids. Only a few reports provide objective information on these two challenges.^61,82,98,102^*In vivo* like conditions can imply the physical conditions of cells in an organism that can simulate the 3D cellular structure and its microenvironment.^202,203^ Because of mimicking the multicellular structure and ECM synthesis, spheroids can provide more realistic *in vivo* conditions than a 2D cell culture.^27^ Furthermore, the studies show that spheroids possess a level of protein and gene expressions, which are similar to those *in vivo*.^14^ The cellular microenvironment, on the other hand, is a very dynamic and complex structure both biomechanically and biochemically and consists of ECM, fluid flow, biomolecular gradients, and other cell types.^204^ Although it is quite challenging to imitate such a complex structure *in vitro*, it is necessary to obtain physiologically more realistic 3D structures consisting of well-defined spatial and temporally controlled cells.^205^ When the studies presented in this review article are evaluated in this context, few articles used biomaterials, such as alginate, PEG, and collagen, to better mimic the biomechanical and biochemical microenvironment in spheroid cultures composed of cancer/epithelial/endothelial cells.^65,67,72,73,75,77,88,92,106^ These biomaterials can provide enhanced cell–cell and cell–ECM interactions.^19,184^ Moreover, co-cultured cells could be arranged spatially and temporally in spheroids with controlled perfusion.^61,82,98,102^*In vivo* systems are already quite complex, and thus the full mimicry of these systems is still very challenging now. However, organ-on-a-chip technologies used for the spheroid culture that provide various advantages compared to 2D culture could further develop in terms of biomaterial, perfusion, and co-culture perspective to get closer results to those obtained in *in vivo* conditions.

To mimic the physiological conditions better *in vitro*, there should be several design criteria, such as shear force, medium delivery, chip architecture, and cell type and density, that need to be considered in OOC applications containing spheroids. In the OOC system, nutrient distribution, waste removal, and transport of molecules are created by liquid flow. OOC with liquid shear stress provides better biological function and capability compared to static culture systems.^206,207^ Shear stress can also affect the cell cycle, cell differentiation, gene expression, and signaling of molecules in tumor cells.^208,209^ Moreover, the cells with a high tendency to cluster were reported to form spheroids under high shear force.^96^

To simulate the circulation of the vascular tissue, continuous circulation of the fresh culture medium is desired for OOC applications.^210^ There have been several methods proposed for the homogeneous distribution of the culture medium, such as surface-tension-driven flow, syringe and peristaltic pumping, and hydrostatic and osmotic pressure difference.^211^ Hydrostatic pressure is generated with the help of the pressure difference between the inlet and the outlet on a chip. This method provides a suitable medium exchange for a perfusion cell culture system.^212^ Osmotic pumping is based on the usage of permeable membrane and driving agents in different concentrations.^211^ For instance, the perfusion was ensured with polyethylene glycol (PEG) concentration in a controlled manner by using a cellulose membrane for hepatocyte spheroid formation.^213^ On the other hand, syringe pumping has widely been used for perfusion in spheroid-engineering studies as it enables controllable and continuous flow.^194,214^ A peristaltic pump is an active pumping system that enables liquid manipulation with a positive displacement of a flexible conduit.^211^ For example, with use of a peristaltic pump, the constant medium flow was generated for the perfusion of hepatic spheroids.^215^ Pumping systems that have different working principles enable continuous medium circulation in a cell culture, but hydrodynamic and osmotic pumping systems have several limitations, for instance, the requirement of conductive reagents, low flow rate, and pressure. However, syringe and peristaltic pumping systems permit a sufficient flow rate, simple integration, easy control, and rapid response time.^216^

### 5.3 Current Challenges and Future Perspectives

First, chip material is an important element for spheroid formation and OOC applications. Polydimethylsiloxane (PDMS) is the most frequently used material in the fabrication of microfluidic chips because of its low cost, ease of use, transparency, elasticity, biocompatibility, and gas-permeable properties.^217^ In addition, glass-based materials can be used for fabricating microfluidic devices with their optical transparency, chemical inertness, rigidity, and high-temperature resistance features. However, they are insufficient for mass production because of the slow and expensive production process. Hence, new materials can be exploited for spheroid fabrication and its applications. Furthermore, the fabrication of microfluidic chips should be simple and cost-effective to increase microfluidic chip usage. Herein, 3D printing technologies that allow rapid, easy, and low-cost production can be used for the fabrication of microfluidic systems from different materials. These technologies also enable fabrication of transparent and complex geometries in a short time.^218,219^ Meanwhile, design parameters such as fluid shear force, concentration gradient, and dynamic mechanical stress should be improved while mimicking physiological conditions *in vitro* using integrated elements such as pumps, valves, and different actuators. In addition, the integration of these systems can enable automated, highly efficient, and reproducible experiments. Different sensors can also be integrated into the microfluidic chips to monitor different parameters to evaluate spheroid formation and culture in real time. Integrated sensor systems can be used not only to detect the amount of metabolic product in the culture environment but also to simultaneously observe cell behavior, mechanical and electrical stimulation, chemical gradient, pH, and gas changes.^220^ Therefore, the spheroid formation and culture process can be easily controlled and also be adjusted for repeatable experiments. Furthermore, these sensors can be utilized to monitor drug responses of formed spheroids.^221^

Furthermore, an all-encompassing culture medium should be determined to culture spheroids containing more than one cell type. To model an organ truly *in vitro*, the metabolites and microbiome system of this organ need to be integrated on a chip level with spheroid culture. Instead of using cell lines, patient-specific cells can be used to form spheroids for personalized medicine applications. Patient-derived spheroids can be utilized in microfluidic chips to assess drug efficacies for each individual that can be employed for digital twin applications. While constituting these chips, one should also consider the differences between the individuals, such as age, gender, ethnicity, and genetics.

Lastly, the developments in OOC technology can eliminate animal testing in preclinical studies and enable multiple drug testing on a single chip. Similarly, the integration of emerging “artificial intelligence” techniques into microfluidic-based spheroid-engineering methods by controlling spheroid size formation, evaluating new drug candidates, and analyzing drug responses could lead to the development of next-generation tools for effective drug studies.^168,222^ These developments can eliminate animal tests and accelerate preclinical analyses and may lead to “clinical trials-on-a-chip” technology in the future.

The global 3D cell culture market including spheroid engineering is expected to grow from $1.3 billion (U.S. dollars) in 2022 to $2.6 billion by 2027, at a compounded annual growth rate (CAGR) of 15.6% between 2022 and 2027.^223^ Conventional spheroid-engineering methodologies are applied in different products using 24-, 96-, or 384-well microtiter plates, such as Perfecta3D hanging drop plates,^224^ Corning spheroid ULA (Ultra-Low Attachment surface) microplates,^225^ and MicroTissues 3D Petri Dish micromold spheroids.^226^ Moreover, droplet-based 3D bioprinted technology could also be applied for spheroid fabrication.^227,228^ There are also many companies in the market supplying solutions for microfluidic-based 3D cell culture and organ-on-a-chip. For instance, Fluigent has developed microfluidic chips that are used in 3D cell culture to generate an hypoxia environment for tumor cells, endothelium/epithelium barrier, and vascularization.^227^ Creative Biolabs Microfluidics Company supplies 3D cell culture chips. These chips enable a culture of neurons, skin, stomach, intestinal, and kidney cells and also their co-culture.^227^ In addition to these companies, Emulate has improved organ-specific organ-on-a-chip devices for modeling of brain, colon intestine, duodenum intestine, kidney, liver, and lung.^227^ Moreover, Dolomite Microfluidics,^227^ Schott Minifab,^227^ and Droplet Genomics^227^ developed droplet-based and microwell-based microfluidic devices. These commercial devices could possibly be applied for spheroid-engineering applications.

## 6 Conclusion

2D culture systems with monolayer cells are limited by cell–cell and cell–extracellular matrix interactions, and these systems cannot mimic the cell microenvironment. However, 3D culture systems overcome these limitations and equally provide nutrient, gas, and growth factor transport into cells. Spheroids as 3D cell aggregates show similar functions to *in vivo* tissues. These cell aggregates are traditionally fabricated with various techniques such as pellet culture, hanging drop, spinner culture, magnetic levitation, etc. Microfluidic approaches have also been developed for spheroid studies. Compared to conventional spheroid formation methods, microfluidic systems ensure the controlled formation of a spheroid with simple and automated processing steps. Moreover, using spheroids in OOC models allows the easy realization of *in vivo* conditions for disease-modeling and drug-screening fields to understand complex physiologies of tissues and organs. In the field of OOC, studies composed of the production of single tissues or organs have come a long way in the past decade. Especially, the co-culture of different cell types on a single chip can simulate better *in vivo* physiological conditions. However, for multiple tissue studies, a different cell culture medium was necessary for different cell lines. To overcome this bottleneck, the spheroid culture of different cells may be maintained in different microcompartments on a chip, and sensors and actuators may also be integrated to monitor and regulate metabolites in a culture medium. Hence, these developments in spheroid engineering using microfluidics could lead to next-generation tools for accurate disease modeling and treatment.

## Data Availability

The data underlying this study are available from the corresponding author upon reasonable request.

## References

[ref1] Gupta N.; Liu J. R.; Patel B.; Solomon D. E.; Vaidya B.; Gupta V. Microfluidics-based 3D Cell Culture Models: Utility in Novel Drug Discovery and Delivery Research. Bioeng. Transl. Med. 2016, 1, 63 10.1002/btm2.10013. 29313007 PMC5689508

[ref2] Breslin S.; O’Driscoll L. Three-Dimensional Cell Culture: The Missing Link in Drug Discovery. Drug Discovery Today 2013, 18, 240 10.1016/j.drudis.2012.10.003. 23073387

[ref3] Hudu S. A.; Alshrari A. S.; Syahida A.; Sekawi Z. Cell Culture, Technology: Enhancing the Culture of Diagnosing Human Diseases. Journal of Clinical and Diagnostic Research. 2016, 10.7860/JCDR/2016/15837.7460. PMC4843260 27134874

[ref4] Duval K.; Grover H.; Han L. H.; Mou Y.; Pegoraro A. F.; Fredberg J.; Chen Z. Modeling Physiological Events in 2D vs. 3D Cell Culture. Physiology. 2017, 32, 266 10.1152/physiol.00036.2016. 28615311 PMC5545611

[ref5] Ravi M.; Paramesh V.; Kaviya S. R.; Anuradha E.; Solomon F. D. P. 3D Cell Culture Systems: Advantages and Applications. J. Cell. Physiol. 2015, 230, 16 10.1002/jcp.24683. 24912145

[ref6] Stacey G. Current Developments in Cell Culture Technology. Adv. Exp. Med. Biol. 2012, 745, 1 10.1007/978-1-4614-3055-1_1. 22437809

[ref7] Kapałczyńska M.; Kolenda T.; Przybyła W.; Zajaczkowska M.; Teresiak A.; Filas V.; Ibbs M.; Bliźniak R.; Łuczewski Ł.; Lamperska K. 2D and 3D Cell Cultures - a Comparison of Different Types of Cancer Cell Cultures. Arch. Med. Sci. 2018, 14, 910–919. 10.5114/aoms.2016.63743. 30002710 PMC6040128

[ref8] Antoni D.; Burckel H.; Josset E.; Noel G. Three-Dimensional Cell Culture: A Breakthrough in Vivo. International Journal of Molecular Sciences. 2015, 16, 5517–5527. 10.3390/ijms16035517. 25768338 PMC4394490

[ref9] Justice B. A.; Badr N. A.; Felder R. A. 3D Cell Culture Opens New Dimensions in Cell-Based Assays. Drug Discovery Today. 2009, 14, 102 10.1016/j.drudis.2008.11.006. 19049902

[ref10] Kim J. B. Three-Dimensional Tissue Culture Models in Cancer Biology. Seminars in Cancer Biology. 2005, 15, 365–377. 10.1016/j.semcancer.2005.05.002. 15975824

[ref11] Page H.; Flood P.; Reynaud E. G. Three-Dimensional Tissue Cultures: Current Trends and Beyond. Cell and Tissue Research. 2013, 352, 123 10.1007/s00441-012-1441-5. 22729488

[ref12] Chen Q.; Wang Y. The Application of Three-Dimensional Cell Culture in Clinical Medicine. Biotechnol. Lett. 2020, 42, 2071 10.1007/s10529-020-03003-y. 32935182

[ref13] Lopa S.; Piraino F.; Talò G.; Mainardi V. L.; Bersini S.; Pierro M.; Zagra L.; Rasponi M.; Moretti M. Microfluidic Biofabrication of 3D Multicellular Spheroids by Modulation of Non-Geometrical Parameters. Front. Bioeng. Biotechnol. 2020, 10.3389/fbioe.2020.00366. PMC7214796 32432090

[ref14] Mehta G.; Hsiao A. Y.; Ingram M.; Luker G. D.; Takayama S. Opportunities and Challenges for Use of Tumor Spheroids as Models to Test Drug Delivery and Efficacy. J. Controlled Release 2012, 164, 192 10.1016/j.jconrel.2012.04.045. PMC3436947 22613880

[ref15] Lee S. H.; Jun B. H. Advances in Dynamic Microphysiological Organ-on-a-Chip: Design Principle and Its Biomedical Application. J. Ind. Eng. Chem. 2019, 71, 65–77. 10.1016/j.jiec.2018.11.041.

[ref16] Decarli M. C.; Amaral R.; dos Santos D. P.; Tofani L. B.; Katayama E.; Rezende R. A.; Silva J. V. L. Da.; Swiech K.; Suazo C. A. T.; Mota C.; Moroni L.; Moraes Â. M. Cell Spheroids as a Versatile Research Platform: Formation Mechanisms, High Throughput Production, Characterization and Applications. Biofabrication 2021, 13 (3), 032002 10.1088/1758-5090/abe6f2. 33592595

[ref17] Kim S.-j.; Kim E. M.; Yamamoto M.; Park H.; Shin H. Engineering Multi-Cellular Spheroids for Tissue Engineering and Regenerative Medicine. Adv. Healthc. Mater. 2020, 9 (23), 2000608 10.1002/adhm.202000608. 32734719

[ref18] Kamatar A.; Gunay G.; Acar H. Natural and Synthetic Biomaterials for Engineering Multicellular Tumor Spheroids. Polymers (Basel). 2020, 12 (11), 2506 10.3390/polym12112506. 33126468 PMC7692845

[ref19] Vadivelu R. K.; Kamble H.; Shiddiky M. J. A.; Nguyen N. T. Microfluidic Technology for the Generation of Cell Spheroids and Their Applications. Micromachines. 2017, 8, 94 10.3390/mi8040094.

[ref20] Moshksayan K.; Kashaninejad N.; Warkiani M. E.; Lock J. G.; Moghadas H.; Firoozabadi B.; Saidi M. S.; Nguyen N. T. Spheroids-on-a-Chip: Recent Advances and Design Considerations in Microfluidic Platforms for Spheroid Formation and Culture. Sensors and Actuators, B: Chemical. 2018, 263, 151 10.1016/j.snb.2018.01.223.

[ref21] Coluccio M. L.; Perozziello G.; Malara N.; Parrotta E.; Zhang P.; Gentile F.; Limongi T.; Raj P. M.; Cuda G.; Candeloro P.; Di Fabrizio E. Microfluidic Platforms for Cell Cultures and Investigations. Microelectron. Eng. 2019, 208, 14–28. 10.1016/j.mee.2019.01.004.

[ref22] Shao C.; Chi J.; Zhang H.; Fan Q.; Zhao Y.; Ye F. Development of Cell Spheroids by Advanced Technologies. Advanced Materials Technologies. 2020, 5, 2000183 10.1002/admt.202000183.

[ref23] Shen H.; Cai S.; Wu C.; Yang W.; Yu H.; Liu L. Recent Advances in Three-Dimensional Multicellular Spheroid Culture and Future Development. Micromachines 2021, 12 (1), 96 10.3390/mi12010096. 33477508 PMC7831097

[ref24] Hoarau-Véchot J.; Rafii A.; Touboul C.; Pasquier J. Halfway between 2D and Animal Models: Are 3D Cultures the Ideal Tool to Study Cancer-Microenvironment Interactions?. International Journal of Molecular Sciences. 2018, 19, 181 10.3390/ijms19010181. 29346265 PMC5796130

[ref25] Lv D.; Hu Z.; Lu L.; Lu H.; Xu X. Three-Dimensional Cell Culture: A Powerful Tool in Tumor Research and Drug Discovery. Oncology Letters. 2017, 10.3892/ol.2017.7134. PMC5754907 29344128

[ref26] Oliveira M. B.; Neto A. I.; Correia C. R.; Rial-Hermida M. I.; Alvarez-Lorenzo C.; Mano J. F. Superhydrophobic Chips for Cell Spheroids High-Throughput Generation and Drug Screening. ACS Appl. Mater. Interfaces 2014, 6, 9488 10.1021/am5018607. 24865973

[ref27] Lin R. Z.; Chang H. Y. Recent Advances in Three-Dimensional Multicellular Spheroid Culture for Biomedical Research. Biotechnology Journal. 2008, 3, 1172 10.1002/biot.200700228. 18566957

[ref28] Kyffin J. A.; Cox C. R.; Leedale J.; Colley H. E.; Murdoch C.; Mistry P.; Webb S. D.; Sharma P. Preparation of Primary Rat Hepatocyte Spheroids Utilizing the Liquid-Overlay Technique. Curr. Protoc. Toxicol. 2019, 10.1002/cptx.87. PMC9285795 31529797

[ref29] Achilli T. M.; Meyer J.; Morgan J. R. Advances in the Formation, Use and Understanding of Multi-Cellular Spheroids. Expert Opinion on Biological Therapy. 2012, 12, 1347 10.1517/14712598.2012.707181. 22784238 PMC4295205

[ref30] Marrella A.; Fedi A.; Varani G.; Vaccari I.; Fato M.; Firpo G.; Guida P.; Aceto N.; Scaglione S. High Blood Flow Shear Stress Values Are Associated with Circulating Tumor Cells Cluster Disaggregation in a Multi-Channel Microfluidic Device. PLoS One 2021, 16 (1), e0245536 10.1371/journal.pone.0245536. 33444361 PMC7808575

[ref31] Fattahi P.; Rahimian A.; Slama M. Q.; Gwon K.; Gonzalez-Suarez A. M.; Wolf J.; Baskaran H.; Duffy C. D.; Stybayeva G.; Peterson Q. P.; Revzin A. Core-Shell Hydrogel Microcapsules Enable Formation of Human Pluripotent Stem Cell Spheroids and Their Cultivation in a Stirred Bioreactor. Sci. Rep. 2021, 10.1038/s41598-021-85786-2. PMC8010084 33785778

[ref32] Phelan M. A.; Gianforcaro A. L.; Gerstenhaber J. A.; Lelkes P. I. An Air Bubble-Isolating Rotating Wall Vessel Bioreactor for Improved Spheroid/Organoid Formation. Tissue Eng. - Part C Methods 2019, 25, 479 10.1089/ten.tec.2019.0088. 31328683 PMC6686703

[ref33] Massai D.; Isu G.; Madeddu D.; Cerino G.; Falco A.; Frati C.; Gallo D.; Deriu M. A.; Falvo D’Urso Labate G.; Quaini F.; Audenino A.; Morbiducci U. A Versatile Bioreactor for Dynamic Suspension Cell Culture. Application to the Culture of Cancer Cell Spheroids. PLoS One 2016, 11 (5), e0154610 10.1371/journal.pone.0154610. 27144306 PMC4856383

[ref34] Raghavan S.; Mehta P.; Horst E. N.; Ward M. R.; Rowley K. R.; Mehta G. Comparative Analysis of Tumor Spheroid Generation Techniques for Differential in Vitro Drug Toxicity. Oncotarget 2016, 7 (13), 16948–16961. 10.18632/oncotarget.7659. 26918944 PMC4941362

[ref35] Ko E.; Poon M. L. S.; Park E.; Cho Y.; Shin J. H. Engineering 3D Cortical Spheroids for an in Vitro Ischemic Stroke Model. ACS Biomater. Sci. Eng. 2021, 7 (8), 3845–3860. 10.1021/acsbiomaterials.1c00406. 34275269

[ref36] Vinci M.; Gowan S.; Boxall F.; Patterson L.; Zimmermann M.; Court W.; Lomas C.; Mendiola M.; Hardisson D.; Eccles S. A. Advances in Establishment and Analysis of Three-Dimensional Tumor Spheroid-Based Functional Assays for Target Validation and Drug Evaluation. BMC Biol. 2012, 10 ( (March), ). 10.1186/1741-7007-10-29. PMC3349530 22439642

[ref37] Shi W.; Kwon J.; Huang Y.; Tan J.; Uhl C. G.; He R.; Zhou C.; Liu Y. Facile Tumor Spheroids Formation in Large Quantity with Controllable Size and High Uniformity. Sci. Rep. 2018, 8 (1), 1–9. 10.1038/s41598-018-25203-3. 29717201 PMC5931581

[ref38] Vadivelu R. K.; Ooi C. H.; Yao R. Q.; Tello Velasquez J.; Pastrana E.; Diaz-Nido J.; Lim F.; Ekberg J. A. K.; Nguyen N. T.; St John J. A. Generation of Three-Dimensional Multiple Spheroid Model of Olfactory Ensheathing Cells Using Floating Liquid Marbles. Sci. Rep. 2015, 10.1038/srep15083. PMC4604460 26462469

[ref39] Li H.; Liu P.; Kaur G.; Yao X.; Yang M. Transparent and Gas-Permeable Liquid Marbles for Culturing and Drug Sensitivity Test of Tumor Spheroids. Adv. Healthc. Mater. 2017, 6, 1700185 10.1002/adhm.201700185. 28426154

[ref40] Chen M.; Shah M. P.; Shelper T. B.; Nazareth L.; Barker M.; Tello Velasquez J.; Ekberg J. A. K.; Vial M. L.; St John J. A. Naked Liquid Marbles: A Robust Three-Dimensional Low-Volume Cell-Culturing System. ACS Appl. Mater. Interfaces 2019, 11, 9814 10.1021/acsami.8b22036. 30724549

[ref41] Oliveira N. M.; Correia C. R.; Reis R. L.; Mano J. F. Liquid Marbles for High-Throughput Biological Screening of Anchorage-Dependent Cells. Adv. Healthc. Mater. 2015, 4, 264 10.1002/adhm.201400310. 25091700

[ref42] Yaman S.; Anil-Inevi M.; Ozcivici E.; Tekin H. C. Magnetic Force-Based Microfluidic Techniques for Cellular and Tissue Bioengineering. Frontiers in Bioengineering and Biotechnology. 2018, 10.3389/fbioe.2018.00192. PMC6305723 30619842

[ref43] Anil-Inevi M.; Yaman S.; Yildiz A. A.; Mese G.; Yalcin-Ozuysal O.; Tekin H. C.; Ozcivici E. Biofabrication of in Situ Self Assembled 3D Cell Cultures in a Weightlessness Environment Generated Using Magnetic Levitation. Sci. Rep. 2018, 10.1038/s41598-018-25718-9. PMC5940762 29740095

[ref44] Durmus N. G.; Tekin H. C.; Guven S.; Sridhar K.; Arslan Yildiz A.; Calibasi G.; Ghiran I.; Davis R. W.; Steinmetz L. M.; Demirci U. Magnetic Levitation of Single Cells. Proc. Natl. Acad. Sci. U. S. A. 2015, 112, E3661–E3668. 10.1073/pnas.1509250112. 26124131 PMC4507238

[ref45] Anil-Inevi M.; Delikoyun K.; Mese G.; Tekin H. C.; Ozcivici E. Magnetic Levitation Assisted Biofabrication, Culture, and Manipulation of 3D Cellular Structures Using a Ring Magnet Based Setup. Biotechnol. Bioeng. 2021, 118, 4771 10.1002/bit.27941. 34559409

[ref46] Sarigil O.; Anil-Inevi M.; Firatligil-Yildirir B.; Unal Y. C.; Yalcin-Ozuysal O.; Mese G.; Tekin H. C.; Ozcivici E. Scaffold-Free Biofabrication of Adipocyte Structures with Magnetic Levitation. Biotechnol. Bioeng. 2021, 118, 1127 10.1002/bit.27631. 33205833

[ref47] Ino K.; Ito A.; Honda H. Cell Patterning Using Magnetite Nanoparticles and Magnetic Force. Biotechnol. Bioeng. 2007, 97, 1309 10.1002/bit.21322. 17216656

[ref48] Li Y.; Kumacheva E. Hydrogel Microenvironments for Cancer Spheroid Growth and Drug Screening. Science Advances. 2018, 10.1126/sciadv.aas8998. PMC5922799 29719868

[ref49] Costa E. C.; Moreira A. F.; de Melo-Diogo D.; Gaspar V. M.; Carvalho M. P.; Correia I. J. 3D Tumor Spheroids: An Overview on the Tools and Techniques Used for Their Analysis. Biotechnology Advances. 2016, 34, 1427 10.1016/j.biotechadv.2016.11.002. 27845258

[ref50] Kim M.; Mun H.; Sung C. O.; Cho E. J.; Jeon H. J.; Chun S. M.; Jung D. J.; Shin T. H.; Jeong G. S.; Kim D. K.; Choi E. K.; Jeong S. Y.; Taylor A. M.; Jain S.; Meyerson M.; Jang S. J. Patient-Derived Lung Cancer Organoids as in Vitro Cancer Models for Therapeutic Screening. Nat. Commun. 2019, 10.1038/s41467-019-11867-6. PMC6728380 31488816

[ref51] Ruedinger F.; Lavrentieva A.; Blume C.; Pepelanova I.; Scheper T. Hydrogels for 3D Mammalian Cell Culture: A Starting Guide for Laboratory Practice. Appl. Microbiol. Biotechnol. 2015, 99, 623 10.1007/s00253-014-6253-y. 25432676

[ref52] Sauty B.; Santesarti G.; Fleischhammer T.; Lindner P.; Lavrentieva A.; Pepelanova I.; Marino M. Enabling Technologies for Obtaining Desired Stiffness Gradients in GelMA Hydrogels Constructs. Macromol. Chem. Phys. 2022, 223 (2), 2100326 10.1002/macp.202100326.

[ref53] van Duinen V.; Trietsch S. J.; Joore J.; Vulto P.; Hankemeier T. Microfluidic 3D Cell Culture: From Tools to Tissue Models. Current Opinion in Biotechnology. 2015, 35, 118 10.1016/j.copbio.2015.05.002. 26094109

[ref54] Wu X.; Su J.; Wei J.; Jiang N.; Ge X. Recent Advances in Three-Dimensional Stem Cell Culture Systems and Applications. Stem Cells International. 2021, 2021, 1 10.1155/2021/9477332. PMC8523294 34671401

[ref55] Inamdar N. K.; Borenstein J. T. Microfluidic Cell Culture Models for Tissue Engineering. Current Opinion in Biotechnology. 2011, 22, 681 10.1016/j.copbio.2011.05.512. 21723720

[ref56] Harink B.; Le Gac S.; Truckenmüller R.; Van Blitterswijk C.; Habibovic P. Regeneration-on-a-Chip? The Perspectives on Use of Microfluidics in Regenerative Medicine. Lab on a Chip. 2013, 13, 3512 10.1039/c3lc50293g. 23877890

[ref57] Damiati S.; Kompella U. B.; Damiati S. A.; Kodzius R. Microfluidic Devices for Drug Delivery Systems and Drug Screening. Genes. 2018, 9, 103 10.3390/genes9020103. 29462948 PMC5852599

[ref58] Li Y.; Li D.; Zhao P.; Nandakumar K.; Wang L.; Song Y. Microfluidics-Based Systems in Diagnosis of Alzheimer’s Disease and Biomimetic Modeling. Micromachines. 2020, 11, 787 10.3390/mi11090787. 32825153 PMC7569794

[ref59] Lee S. A.; No D. Y.; Kang E.; Ju J.; Kim D. S.; Lee S. H. Spheroid-Based Three-Dimensional Liver-on-a-Chip to Investigate Hepatocyte-Hepatic Stellate Cell Interactions and Flow Effects. Lab Chip 2013, 13, 3529 10.1039/c3lc50197c. 23657720

[ref60] Li X.; Valadez A. V.; Zuo P.; Nie Z. Microfluidic 3D Cell Culture: Potential Application for Tissue-Based Bioassays. Bioanalysis. 2012, 4, 1509 10.4155/bio.12.133. 22793034 PMC3909686

[ref61] Sart S.; Tomasi R. F. X.; Amselem G.; Baroud C. N. Multiscale Cytometry and Regulation of 3D Cell Cultures on a Chip. Nat. Commun. 2017, 10.1038/s41467-017-00475-x. PMC5589863 28883466

[ref62] Alessandri K.; Sarangi B. R.; Gurchenkov V. V.; Sinha B.; Kießling T. R.; Fetler L.; Rico F.; Scheuring S.; Lamaze C.; Simon A.; Geraldo S.; Vignjević D.; Doméjean H.; Rolland L.; Funfak A.; Bibette J.; Bremond N.; Nassoy P. Cellular Capsules as a Tool for Multicellular Spheroid Production and for Investigating the Mechanics of Tumor Progression in Vitro. Proc. Natl. Acad. Sci. U. S. A. 2013, 110, 14843 10.1073/pnas.1309482110. 23980147 PMC3773746

[ref63] Wang Y.; Wang J. Mixed Hydrogel Bead-Based Tumor Spheroid Formation and Anticancer Drug Testing. Analyst 2014, 139, 2449 10.1039/C4AN00015C. 24699505

[ref64] Chan H. F.; Zhang Y.; Ho Y. P.; Chiu Y. L.; Jung Y.; Leong K. W. Rapid Formation of Multicellular Spheroids in Double-Emulsion Droplets with Controllable Microenvironment. Sci. Rep. 2013, 10.1038/srep03462. PMC3857570 24322507

[ref65] Sabhachandani P.; Sarkar S.; Mckenney S.; Ravi D.; Evens A. M.; Konry T. Microfluidic Assembly of Hydrogel-Based Immunogenic Tumor Spheroids for Evaluation of Anticancer Therapies and Biomarker Release. J. Controlled Release 2019, 295, 21 10.1016/j.jconrel.2018.12.010. PMC6396303 30550941

[ref66] Kim C.; Chung S.; Kim Y. E.; Lee K. S.; Lee S. H.; Oh K. W.; Kang J. Y. Generation of Core-Shell Microcapsules with Three-Dimensional Focusing Device for Efficient Formation of Cell Spheroid. Lab Chip 2011, 11, 246 10.1039/C0LC00036A. 20967338

[ref67] Sun Q.; Tan S. H.; Chen Q.; Ran R.; Hui Y.; Chen D.; Zhao C. X. Microfluidic Formation of Coculture Tumor Spheroids with Stromal Cells As a Novel 3D Tumor Model for Drug Testing. ACS Biomater. Sci. Eng. 2018, 4, 4425 10.1021/acsbiomaterials.8b00904. 33418835

[ref68] Kamperman T.; Henke S.; Visser C. W.; Karperien M.; Leijten J. Centering Single Cells in Microgels via Delayed Crosslinking Supports Long-Term 3D Culture by Preventing Cell Escape. Small 2017, 13, 1603711 10.1002/smll.201603711. 28452168

[ref69] Lee J. M.; Choi J. W.; Ahrberg C. D.; Choi H. W.; Ha J. H.; Mun S. G.; Mo S. J.; Chung B. G. Generation of Tumor Spheroids Using a Droplet-Based Microfluidic Device for Photothermal Therapy. Microsystems Nanoeng. 2020, 10.1038/s41378-020-0167-x. PMC8433304 34567663

[ref70] Imaninezhad M.; Hill L.; Kolar G.; Vogt K.; Zustiak S. P. Templated Macroporous Polyethylene Glycol Hydrogels for Spheroid and Aggregate Cell Culture. Bioconjugate Chem. 2019, 30, 34 10.1021/acs.bioconjchem.8b00596. 30562006

[ref71] Hou Y.; Xie W.; Achazi K.; Cuellar-Camacho J. L.; Melzig M. F.; Chen W.; Haag R. Injectable Degradable PVA Microgels Prepared by Microfluidic Technology for Controlled Osteogenic Differentiation of Mesenchymal Stem Cells. Acta Biomater. 2018, 77, 28 10.1016/j.actbio.2018.07.003. 29981495

[ref72] Sabhachandani P.; Motwani V.; Cohen N.; Sarkar S.; Torchilin V.; Konry T. Generation and Functional Assessment of 3D Multicellular Spheroids in Droplet Based Microfluidics Platform. Lab Chip 2016, 16, 497 10.1039/C5LC01139F. 26686985 PMC4834071

[ref73] Langer K.; Joensson H. N. Rapid Production and Recovery of Cell Spheroids by Automated Droplet Microfluidics. SLAS Technol. 2020, 25, 111 10.1177/2472630319877376. 31561747

[ref74] Eydelnant I. A.; Betty Li B.; Wheeler A. R. Microgels On-Demand. Nat. Commun. 2014, 10.1038/ncomms4355. 24566526

[ref75] Au S. H.; Chamberlain M. D.; Mahesh S.; Sefton M. V.; Wheeler A. R. Hepatic Organoids for Microfluidic Drug Screening. Lab Chip 2014, 14, 3290 10.1039/C4LC00531G. 24984750

[ref76] Aijian A. P.; Garrell R. L. Digital Microfluidics for Automated Hanging Drop Cell Spheroid Culture. J. Lab. Autom. 2015, 20, 283 10.1177/2211068214562002. 25510471

[ref77] Lee J. M.; Park D. Y.; Yang L.; Kim E. J.; Ahrberg C. D.; Lee K. B.; Chung B. G. Generation of Uniform-Sized Multicellular Tumor Spheroids Using Hydrogel Microwells for Advanced Drug Screening. Sci. Rep. 2018, 10.1038/s41598-018-35216-7. PMC6249215 30464248

[ref78] Chen Z.; Kheiri S.; Gevorkian A.; Young E. W. K.; Andre V.; Deisenroth T.; Kumacheva E. Microfluidic Arrays of Dermal Spheroids: A Screening Platform for Active Ingredients of Skincare Products. Lab Chip 2021, 21, 3952 10.1039/D1LC00619C. 34636823

[ref79] Gong X.; Lin C.; Cheng J.; Su J.; Zhao H.; Liu T.; Wen X.; Zhao P. Generation of Multicellular Tumor Spheroids with Microwell-Based Agarose Scaffolds for Drug Testing. PLoS One 2015, 10, e0130348 10.1371/journal.pone.0130348. 26090664 PMC4474551

[ref80] Azizipour N.; Avazpour R.; Weber M. H.; Sawan M.; Ajji A.; Rosenzweig D. H. Uniform Tumor Spheroids on Surface-Optimized Microfluidic Biochips for Reproducible Drug Screening and Personalized Medicine. Micromachines 2022, 13 (4), 587 10.3390/mi13040587. 35457892 PMC9028696

[ref81] Mulholland T.; McAllister M.; Patek S.; Flint D.; Underwood M.; Sim A.; Edwards J.; Zagnoni M. Drug Screening of Biopsy-Derived Spheroids Using a Self-Generated Microfluidic Concentration Gradient. Sci. Rep. 2018, 10.1038/s41598-018-33055-0. PMC6168499 30279484

[ref82] Huang C. K.; Paylaga G. J.; Bupphathong S.; Lin K. H. Spherical Microwell Arrays for Studying Single Cells and Microtissues in 3D Confinement. Biofabrication 2020, 12, 025016 10.1088/1758-5090/ab6eda. 31974317

[ref83] Khot M. I.; Levenstein M. A.; de Boer G. N.; Armstrong G.; Maisey T.; Svavarsdottir H. S.; Andrew H.; Perry S. L.; Kapur N.; Jayne D. G. Characterising a PDMS Based 3D Cell Culturing Microfluidic Platform for Screening Chemotherapeutic Drug Cytotoxic Activity. Sci. Rep. 2020, 10.1038/s41598-020-72952-1. PMC7522244 32985610

[ref84] Lim W.; Park S. A Microfluidic Spheroid Culture Device with a Concentration Gradient Generator for High-Throughput Screening of Drug Efficacy. Molecules 2018, 23, 3355 10.3390/molecules23123355. 30567363 PMC6321514

[ref85] Pitingolo G.; Nizard P.; Riaud A.; Taly V. Beyond the on/off Chip Trade-off: A Reversibly Sealed Microfluidic Platform for 3D Tumor Microtissue Analysis. Sensors Actuators, B Chem. 2018, 274, 393 10.1016/j.snb.2018.07.166.

[ref86] Seyfoori A.; Samiei E.; Jalili N.; Godau B.; Rahmanian M.; Farahmand L.; Majidzadeh-A K.; Akbari M. Self-Filling Microwell Arrays (SFMAs) for Tumor Spheroid Formation. Lab Chip 2018, 18, 3516 10.1039/C8LC00708J. 30357219

[ref87] Bhise N. S; Manoharan V.; Massa S.; Tamayol A.; Ghaderi M.; Miscuglio M.; Lang Q.; Shrike Zhang Y.; Shin S. R.; Calzone G.; Annabi N.; Shupe T. D; Bishop C. E; Atala A.; Dokmeci M. R; Khademhosseini A. A Liver-on-a-Chip Platform with Bioprinted Hepatic Spheroids. Biofabrication 2016, 8, 014101 10.1088/1758-5090/8/1/014101. 26756674

[ref88] Zuchowska A.; Jastrzebska E.; Chudy M.; Dybko A.; Brzozka Z. Advanced 3D Spheroid Culture for Evaluation of Photodynamic Therapy in Microfluidic System. Procedia Engineering 2016, 168, 403 10.1016/j.proeng.2016.11.184.

[ref89] Patra B.; Chen Y. H.; Peng C. C.; Lin S. C.; Lee C. H.; Tung Y. C. A Microfluidic Device for Uniform-Sized Cell Spheroids Formation, Culture, Harvesting and Flow Cytometry Analysis. Biomicrofluidics 2013, 7, 054114 10.1063/1.4824480. 24396525 PMC3808411

[ref90] Fan Y.; Nguyen D. T.; Akay Y.; Xu F.; Akay M. Engineering a Brain Cancer Chip for High-Throughput Drug Screening. Sci. Rep. 2016, 10.1038/srep25062. PMC4858657 27151082

[ref91] Trinh K. T. L.; Le N. X. T.; Lee N. Y. Chitosan-Polydopamine Hydrogel Complex: A Novel Green Adhesion Agent for Reversibly Bonding Thermoplastic Microdevice and Its Application for Cell-Friendly Microfluidic 3D Cell Culture. Lab Chip 2020, 20, 3524 10.1039/D0LC00621A. 32869048

[ref92] Huang Y. L.; Ma Y.; Wu C.; Shiau C.; Segall J. E.; Wu M. Tumor Spheroids under Perfusion within a 3D Microfluidic Platform Reveal Critical Roles of Cell-Cell Adhesion in Tumor Invasion. Sci. Rep. 2020, 10.1038/s41598-020-66528-2. PMC7295764 32541776

[ref93] Zhao L.; Liu Y.; Liu Y.; Zhang M.; Zhang X. Microfluidic Control of Tumor and Stromal Cell Spheroids Pairing and Merging for Three-Dimensional Metastasis Study. Anal. Chem. 2020, 92, 7638 10.1021/acs.analchem.0c00408. 32374153

[ref94] Eilenberger C.; Rothbauer M.; Selinger F.; Gerhartl A.; Jordan C.; Harasek M.; Schädl B.; Grillari J.; Weghuber J.; Neuhaus W.; Küpcü S.; Ertl P. A Microfluidic Multisize Spheroid Array for Multiparametric Screening of Anticancer Drugs and Blood-Brain Barrier Transport Properties. Adv. Sci. 2021, 8, 2004856 10.1002/advs.202004856. PMC8188192 34105271

[ref95] Järvinen P.; Bonabi A.; Jokinen V.; Sikanen T. Simultaneous Culturing of Cell Monolayers and Spheroids on a Single Microfluidic Device for Bridging the Gap between 2D and 3D Cell Assays in Drug Research. Adv. Funct. Mater. 2020, 30, 2000479 10.1002/adfm.202000479.

[ref96] Zuchowska A.; Jastrzebska E.; Zukowski K.; Chudy M.; Dybko A.; Brzozka Z. A549 and MRC-5 Cell Aggregation in a Microfluidic Lab-on-a-Chip System. Biomicrofluidics 2017, 11, 024110 10.1063/1.4979104. 28405259 PMC5375957

[ref97] Hsu C.-H.; Chen C.-C. Microfluidic Hanging Drop Chip. TW 201329230 A, 2013.

[ref98] Rodoplu D.; Matahum J. S.; Hsu C.-H. A Microfluidic Hanging Drop-Based Spheroid Co-Culture Platform for Probing Tumor Angiogenesis. Lab Chip 2022, 22, 1275 10.1039/D1LC01177D. 35191460

[ref99] Tung Y. C.; Hsiao A. Y.; Allen S. G.; Torisawa Y. S.; Ho M.; Takayama S. High-Throughput 3D Spheroid Culture and Drug Testing Using a 384 Hanging Drop Array. Analyst 2011, 136, 473 10.1039/C0AN00609B. 20967331 PMC7454010

[ref100] Wu H. W.; Hsiao Y. H.; Chen C. C.; Yet S. F.; Hsu C. H. A Pdms-Based Microfluidic Hanging Drop Chip for Embryoid Body Formation. Molecules 2016, 21, 882 10.3390/molecules21070882. 27399655 PMC6272923

[ref101] Huang S. W.; Tzeng S. C.; Chen J. K.; Sun J. S.; Lin F. H. A Dynamic Hanging-Drop System for Mesenchymal Stem Cell Culture. Int. J. Mol. Sci. 2020, 21, 4298 10.3390/ijms21124298. 32560269 PMC7352343

[ref102] Cho C. Y.; Chiang T. H.; Hsieh L. H.; Yang W. Y.; Hsu H. H.; Yeh C. K.; Huang C. C.; Huang J. H. Development of a Novel Hanging Drop Platform for Engineering Controllable 3D Microenvironments. Front. Cell Dev. Biol. 2020, 10.3389/fcell.2020.00327. PMC7221142 32457907

[ref103] Ganguli A.; Mostafa A.; Saavedra C.; Kim Y.; Le P.; Faramarzi V.; Feathers R. W.; Berger J.; Ramos-Cruz K. P.; Adeniba O.; Diaz G. J. P.; Drnevich J.; Wright C. L.; Hernandez A. G.; Lin W.; Smith A. M.; Kosari F.; Vasmatzis G.; Anastasiadis P. Z.; Bashir R. Three-Dimensional Microscale Hanging Drop Arrays with Geometric Control for Drug Screening and Live Tissue Imaging. Sci. Adv. 2021, 10.1126/sciadv.abc1323. PMC8064630 33893093

[ref104] Liu X.; Lin H.; Song J.; Zhang T.; Wang X.; Huang X.; Zheng C. A Novel Simpledrop Chip for 3d Spheroid Formation and Anti-Cancer Drug Assay. Micromachines 2021, 12, 681 10.3390/mi12060681. 34200752 PMC8230402

[ref105] Sun B.; Zhao Y.; Wu W.; Zhao Q.; Li G. A Superhydrophobic Chip Integrated with an Array of Medium Reservoirs for Long-Term Hanging Drop Spheroid Culture. Acta Biomater. 2021, 135, 234 10.1016/j.actbio.2021.08.006. 34389482

[ref106] Fu C. Y.; Tseng S. Y.; Yang S. M.; Hsu L.; Liu C. H.; Chang H. Y. A Microfluidic Chip with a U-Shaped Microstructure Array for Multicellular Spheroid Formation, Culturing and Analysis. Biofabrication 2014, 6, 015009 10.1088/1758-5082/6/1/015009. 24589876

[ref107] Torisawa Y.-s.; Takagi A.; Nashimoto Y.; Yasukawa T.; Shiku H.; Matsue T. A Multicellular Spheroid Array to Realize Spheroid Formation, Culture, and Viability Assay on a Chip. Biomaterials 2007, 28, 559 10.1016/j.biomaterials.2006.08.054. 16989897

[ref108] He Y.; Huang B.; Rofaani E.; Hu J.; Liu Y.; Pitingolo G.; Wang L.; Shi J.; Aimé C.; Chen Y. Fabrication of Micro-Cages and Caged Tumor Spheroids for Microfluidic Chip-Based Assays. Microelectron. Eng. 2020, 225, 111256 10.1016/j.mee.2020.111256.

[ref109] Yu L.; Chen M. C. W.; Cheung K. C. Droplet-Based Microfluidic System for Multicellular Tumor Spheroid Formation and Anticancer Drug Testing. Lab Chip 2010, 10, 2424 10.1039/c004590j. 20694216

[ref110] Chen Y.; Gao D.; Wang Y.; Lin S.; Jiang Y. A Novel 3D Breast-Cancer-on-Chip Platform for Therapeutic Evaluation of Drug Delivery Systems. Anal. Chim. Acta 2018, 1036, 97 10.1016/j.aca.2018.06.038. 30253842

[ref111] Hardelauf H.; Frimat J. P.; Stewart J. D.; Schormann W.; Chiang Y. Y.; Lampen P.; Franzke J.; Hengstler J. G.; Cadenas C.; Kunz-Schughart L. A.; West J. Microarrays for the Scalable Production of Metabolically Relevant Tumour Spheroids: A Tool for Modulating Chemosensitivity Traits. Lab Chip 2011, 11, 419 10.1039/C0LC00089B. 21079873

[ref112] Barisam M.; Niavol F. R.; Kinj M. A.; Saidi M. S.; Ghanbarian H.; Kashaninejad N. Enrichment of Cancer Stem-like Cells by Controlling Oxygen, Glucose and Fluid Shear Stress in a Microfluidic Spheroid Culture Device. J. Sci. Adv. Mater. Devices 2022, 7, 100439 10.1016/j.jsamd.2022.100439.

[ref113] Dornhof J.; Kieninger J.; Muralidharan H.; Maurer J.; Urban G. A.; Weltin A. Oxygen and Lactate Monitoring in 3D Breast Cancer Organoid Culture with Sensor-Integrated Microfluidic Platform. 21st Int. Conf. Solid-State Sensors, Actuators Microsystems, TRANSDUCERS 2021 2021, (June), , 703–706 10.1109/Transducers50396.2021.9495557

[ref114] Chen B.; Wu Y.; Ao Z.; Cai H.; Nunez A.; Liu Y.; Foley J.; Nephew K.; Lu X.; Guo F. High-Throughput Acoustofluidic Fabrication of Tumor Spheroids. Lab Chip 2019, 19, 1755 10.1039/C9LC00135B. 30918934

[ref115] Wu Z.; Chen B.; Wu Y.; Xia Y.; Chen H.; Gong Z.; Hu H.; Ding Z.; Guo S. Scaffold-Free Generation of Heterotypic Cell Spheroids Using Acoustofluidics. Lab Chip 2021, 21 (18), 3498–3508. 10.1039/D1LC00496D. 34346468

[ref116] Sebastian A.; Buckle A. M.; Markx G. H. Formation of Multilayer Aggregates of Mammalian Cells by Dielectrophoresis. J. Micromechanics Microengineering 2006, 16, 1769 10.1088/0960-1317/16/9/003.

[ref117] Yasukawa T.; Morishima A.; Suzuki M.; Yoshioka J.; Yoshimoto K.; Mizutani F. Rapid Formation of Aggregates with Uniform Numbers of Cells Based on Three-Dimensional Dielectrophoresis. Anal. Sci. 2019, 35, 895 10.2116/analsci.19P074. 31006719

[ref118] Chong D. T.; Liu X. S.; Ma H. J.; Huang G. Y.; Han Y. L.; Cui X. Y.; Yan J. J.; Xu F. Advances in Fabricating Double-Emulsion Droplets and Their Biomedical Applications. Microfluidics and Nanofluidics. 2015, 19, 1071 10.1007/s10404-015-1635-8.

[ref119] Qu F.; Zhao S.; Cheng G.; Rahman H.; Xiao Q.; Chan R. W. Y.; Ho Y. P. Double Emulsion-Pretreated Microwell Culture for the in Vitro Production of Multicellular Spheroids and Their in Situ Analysis. Microsystems Nanoeng. 2021, 10.1038/s41378-021-00267-w. PMC8433470 34567752

[ref120] Zhan Z.; Liu Z.; Nan H.; Li J.; Xie Y.; Hu C. Heterogeneous Spheroids with Tunable Interior Morphologies by Droplet-Based Microfluidics. Biofabrication 2022, 14 (2), 025024 10.1088/1758-5090/ac5e12. 35290971

[ref121] Sharma S.; Srisa-Art M.; Scott S.; Asthana A.; Cass A. Droplet-Based Micro Fluidics. Methods Mol. Biol. 2013, 949, 207 10.1007/978-1-62703-134-9_15. 23329446

[ref122] Sartipzadeh O.; Naghib S. M.; Seyfoori A.; Rahmanian M.; Fateminia F. S. Controllable Size and Form of Droplets in Microfluidic-Assisted Devices: Effects of Channel Geometry and Fluid Velocity on Droplet Size. Mater. Sci. Eng., C 2020, 109, 110606 10.1016/j.msec.2019.110606. 32228988

[ref123] Panhwar M. H.; Czerwinski F.; Dabbiru V. A. S.; Komaragiri Y.; Fregin B.; Biedenweg D.; Nestler P.; Pires R. H.; Otto O. High-Throughput Cell and Spheroid Mechanics in Virtual Fluidic Channels. Nat. Commun. 2020, 10.1038/s41467-020-15813-9. PMC7198589 32366850

[ref124] Marín A. G.; Campo-Cortés F.; Gordillo J. M. Generation of Micron-Sized Drops and Bubbles through Viscous Coflows. Colloids Surfaces A Physicochem. Eng. Asp. 2009, 344, 2 10.1016/j.colsurfa.2008.09.033.

[ref125] Lashkaripour A.; Rodriguez C.; Mehdipour N.; Mardian R.; McIntyre D.; Ortiz L.; Campbell J.; Densmore D. Machine Learning Enables Design Automation of Microfluidic Flow-Focusing Droplet Generation. Nat. Commun. 2021, 10.1038/s41467-020-20284-z. PMC7782806 33397940

[ref126] Lee D.; Cha C. The Combined Effects of Co-Culture and Substrate Mechanics on 3d Tumor Spheroid Formation within Microgels Prepared via Flow-Focusing Microfluidic Fabrication. Pharmaceutics 2018, 10, 229 10.3390/pharmaceutics10040229. 30428559 PMC6321249

[ref127] Choi K.; Ng A. H. C.; Fobel R.; Wheeler A. R. Digital Microfluidics. Annual Review of Analytical Chemistry. 2012, 5, 413 10.1146/annurev-anchem-062011-143028. 22524226

[ref128] Hong J.; Kim Y. K.; Won D. J.; Kim J.; Lee S. J. Three-Dimensional Digital Microfluidic Manipulation of Droplets in Oil Medium. Sci. Rep. 2015, 10.1038/srep10685. PMC4451554 26033440

[ref129] Kothamachu V. B.; Zaini S.; Muffatto F. Role of Digital Microfluidics in Enabling Access to Laboratory Automation and Making Biology Programmable. SLAS Technology. 2020, 25, 411 10.1177/2472630320931794. 32584152

[ref130] Hwang Y. S.; Kim J.; Yoon H. J.; Kang J. I.; Park K. H.; Bae H. Microwell-Mediated Cell Spheroid Formation and Its Applications. Macromolecular Research. 2018, 26, 1 10.1007/s13233-018-6002-7.

[ref131] Kim D.; Kim K.; Park J. Y. Novel Microwell with a Roof Capable of Buoyant Spheroid Culture. Lab Chip 2021, 21, 1974 10.1039/D0LC01295E. 34008588

[ref132] Charnley M.; Textor M.; Khademhosseini A.; Lutolf M. P. Integration Column: Microwell Arrays for Mammalian Cell Culture. Integr. Biol. 2009, 1, 625 10.1039/b918172p. 20027371

[ref133] Pedde R. D.; Mirani B.; Navaei A.; Styan T.; Wong S.; Mehrali M.; Thakur A.; Mohtaram N. K.; Bayati A.; Dolatshahi-Pirouz A.; Nikkhah M.; Willerth S. M.; Akbari M. Emerging Biofabrication Strategies for Engineering Complex Tissue Constructs. Adv. Mater. 2017, 29, 1606061 10.1002/adma.201606061. 28370405

[ref134] Manzoor A. A.; Romita L.; Hwang D. K. A Review on Microwell and Microfluidic Geometric Array Fabrication Techniques and Its Potential Applications in Cellular Studies. Can. J. Chem. Eng. 2021, 99, 61 10.1002/cjce.23875.

[ref135] Rousset N.; Monet F.; Gervais T. Simulation-Assisted Design of Microfluidic Sample Traps for Optimal Trapping and Culture of Non-Adherent Single Cells, Tissues, and Spheroids. Sci. Rep. 2017, 10.1038/s41598-017-00229-1. PMC5428016 28325895

[ref136] Rismani Yazdi S.; Shadmani A.; Bürgel S. C.; Misun P. M.; Hierlemann A.; Frey O. Adding the “heart” to Hanging Drop Networks for Microphysiological Multi-Tissue Experiments. Lab Chip 2015, 15, 4138 10.1039/C5LC01000D. 26401602 PMC5424877

[ref137] Suryaprakash R. T. C.; Kujan O.; Shearston K.; Farah C. S. Three-Dimensional Cell Culture Models to Investigate Oral Carcinogenesis: A Scoping Review. International Journal of Molecular Sciences. 2020, 21, 9520 10.3390/ijms21249520. 33327663 PMC7765087

[ref138] Shen H.; Cai S.; Wu C.; Yang W.; Yu H.; Liu L. Recent Advances in Three-Dimensional Multicellular Spheroid Culture and Future Development. Micromachines. 2021, 12, 96 10.3390/mi12010096. 33477508 PMC7831097

[ref139] Barisam M.; Saidi M. S.; Kashaninejad N.; Vadivelu R.; Nguyen N. T. Numerical Simulation of the Behavior of Toroidal and Spheroidal Multicellular Aggregates in Microfluidic Devices with Microwell and U-Shaped Barrier. Micromachines 2017, 8, 358 10.3390/mi8120358. 30400548 PMC6187926

[ref140] Behroodi E.; Latifi H.; Bagheri Z.; Ermis E.; Roshani S.; Salehi Moghaddam M. A Combined 3D Printing/CNC Micro-Milling Method to Fabricate a Large-Scale Microfluidic Device with the Small Size 3D Architectures: An Application for Tumor Spheroid Production. Sci. Rep. 2020, 10.1038/s41598-020-79015-5. PMC7747638 33335148

[ref141] Torisawa Y. S.; Mosadegh B.; Luker G. D.; Morell M.; O’Shea K. S.; Takayama S. Microfluidic Hydrodynamic Cellular Patterning for Systematic Formation of Co-Culture Spheroids. Integr. Biol. 2009, 1, 649 10.1039/b915965g. PMC2825702 20027373

[ref142] Wang T.; Green R.; Nair R. R.; Howell M.; Mohapatra S.; Guldiken R.; Mohapatra S. S. Surface Acoustic Waves (SAW)-Based Biosensing for Quantification of Cell Growth in 2D and 3D Cultures. Sensors (Switzerland) 2015, 15, 32045 10.3390/s151229909. PMC4721826 26703604

[ref143] Abdallat R. G.; Ahmad Tajuddin A. S.; Gould D. H.; Hughes M. P.; Fatoyinbo H. O.; Labeed F. H. Process Development for Cell Aggregate Arrays Encapsulated in a Synthetic Hydrogel Using Negative Dielectrophoresis. Electrophoresis 2013, 34, 1059 10.1002/elps.201200459. 23436271

[ref144] Petta D.; Basoli V.; Pellicciotta D.; Tognato R.; Barcik J.; Arrigoni C.; Bella E. D.; Armiento A. R.; Candrian C.; Richards R. G.; Alini M.; Moretti M.; Eglin D.; Serra T. Sound-Induced Morphogenesis of Multicellular Systems for Rapid Orchestration of Vascular Networks. 2021, 13, 015004 10.1088/1758-5090/abbb9c. 32977317

[ref145] Chen P.; Guven S.; Usta O. B.; Yarmush M. L.; Demirci U. Biotunable Acoustic Node Assembly of Organoids.. Adv. Healthcare Mater. 2015, 4, 1937 10.1002/adhm.201500279. PMC4731612 26149464

[ref146] Guex A. G.; Di Marzio N.; Eglin D.; Alini M.; Serra T. The Waves That Make the Pattern: A Review on Acoustic Manipulation in Biomedical Research. Materials Today Bio. 2021, 10, 100110 10.1016/j.mtbio.2021.100110. PMC8094912 33997761

[ref147] Prasad S.; Zhang X.; Yang M.; Ni Y.; Parpura V.; Ozkan C. S.; Ozkan M. Separation of Individual Neurons Using Dielectrophoretic Alternative Current Fields. J. Neurosci. Methods 2004, 135, 79 10.1016/j.jneumeth.2003.12.007. 15020092

[ref148] Schneider S.; Gruner D.; Richter A.; Loskill P. Membrane Integration into PDMS-Free Microfluidic Platforms for Organ-on-Chip and Analytical Chemistry Applications. Lab on a Chip. 2021, 21, 1866 10.1039/D1LC00188D. 33949565

[ref149] Azizgolshani H.; Coppeta J. R.; Vedula E. M.; Marr E. E.; Cain B. P.; Luu R. J.; Lech M. P.; Kann S. H.; Mulhern T. J.; Tandon V.; Tan K.; Haroutunian N. J.; Keegan P.; Rogers M.; Gard A. L.; Baldwin K. B.; de Souza J. C.; Hoefler B. C.; Bale S. S.; Kratchman L. B.; Zorn A.; Patterson A.; Kim E. S.; Petrie T. A.; Wiellette E. L.; Williams C.; Isenberg B. C.; Charest J. L. High-Throughput Organ-on-Chip Platform with Integrated Programmable Fluid Flow and Real-Time Sensing for Complex Tissue Models in Drug Development Workflows. Lab Chip 2021, 21, 1454 10.1039/D1LC00067E. 33881130

[ref150] Essaouiba A.; Jellali R.; Shinohara M.; Scheidecker B.; Legallais C.; Sakai Y.; Leclerc E. Analysis of the Behavior of 2D Monolayers and 3D Spheroid Human Pancreatic Beta Cells Derived from Induced Pluripotent Stem Cells in a Microfluidic Environment. J. Biotechnol. 2021, 330, 45 10.1016/j.jbiotec.2021.02.009. 33617908

[ref151] Bovard D.; Iskandar A.; Luettich K.; Hoeng J.; Peitsch M. C. Organs-on-a-Chip. Toxicol. Res. Appl. 2017, 1, 239784731772635 10.1177/2397847317726351.

[ref152] Lancaster M. A.; Huch M. Disease Modelling in Human Organoids. DMM Dis. Model. Mech. 2019, 10.1242/dmm.039347. PMC6679380 31383635

[ref153] Benam K. H.; Dauth S.; Hassell B.; Herland A.; Jain A.; Jang K. J.; Karalis K.; Kim H. J.; MacQueen L.; Mahmoodian R.; Musah S.; Torisawa Y. S.; Van Der Meer A. D.; Villenave R.; Yadid M.; Parker K. K.; Ingber D. E. Engineered in Vitro Disease Models. Annu. Rev. Pathol. Mech. Dis. 2015, 10, 195 10.1146/annurev-pathol-012414-040418. 25621660

[ref154] Li Z.; Hui J.; Yang P.; Mao H. Microfluidic Organ-on-a-Chip System for Disease Modeling and Drug Development.. Biosensors 2022, 12, 370 10.3390/bios12060370. 35735518 PMC9220862

[ref155] Park J.; Wetzel I.; Marriott I.; Dréau D.; D’Avanzo C.; Kim D. Y.; Tanzi R. E.; Cho H. A 3D Human Triculture System Modeling Neurodegeneration and Neuroinflammation in Alzheimer’s Disease. Nat. Neurosci. 2018, 21, 941 10.1038/s41593-018-0175-4. 29950669 PMC6800152

[ref156] Lee H. K.; Velazquez Sanchez C.; Chen M.; Morin P. J.; Wells J. M.; Hanlon E. B.; Xia W. Three Dimensional Human Neuro-Spheroid Model of Alzheimer’s Disease Based on Differentiated Induced Pluripotent Stem Cells. PLoS One 2016, 11, e0163072 10.1371/journal.pone.0163072. 27684569 PMC5042502

[ref157] Park J.; Lee B. K.; Jeong G. S.; Hyun J. K.; Lee C. J.; Lee S. H. Three-Dimensional Brain-on-a-Chip with an Interstitial Level of Flow and Its Application as an in Vitro Model of Alzheimer’s Disease. Lab Chip 2015, 15, 141 10.1039/C4LC00962B. 25317977

[ref158] Li W.; Alazawi W. Non-Alcoholic Fatty Liver Disease. Clin. Med. J. R. Coll. Physicians London 2020, 20, 509 10.7861/clinmed.2020-0696. PMC7539730 32934047

[ref159] Lasli S.; Kim H. J.; Lee K. J.; Suurmond C. A. E.; Goudie M.; Bandaru P.; Sun W.; Zhang S.; Zhang N.; Ahadian S.; Dokmeci M. R.; Lee J.; Khademhosseini A. A Human Liver-on-a-Chip Platform for Modeling Nonalcoholic Fatty Liver Disease. Adv. Biosyst. 2019, 3, 1900104 10.1002/adbi.201900104. PMC7473489 32648699

[ref160] Wang F.; So K. F.; Xiao J.; Wang H. Organ-Organ Communication: The Liver’s Perspective. Theranostics. 2021, 11, 3317 10.7150/thno.55795. 33537089 PMC7847667

[ref161] Bauer S.; Wennberg Huldt C.; Kanebratt K. P.; Durieux I.; Gunne D.; Andersson S.; Ewart L.; Haynes W. G.; Maschmeyer I.; Winter A.; Ämmälä C.; Marx U.; Andersson T. B. Functional Coupling of Human Pancreatic Islets and Liver Spheroids On-a-Chip: Towards a Novel Human Ex Vivo Type 2 Diabetes Model. Sci. Rep. 2017, 10.1038/s41598-017-14815-w. PMC5668271 29097671

[ref162] Herschkowitz J. I.; Behbod F. Human Ductal Carcinoma In Situ: From the Eyes of a Beholder. Journal of Mammary Gland Biology and Neoplasia. 2018, 23, 189 10.1007/s10911-018-9419-x. 30406903 PMC6483100

[ref163] Choi Y.; Hyun E.; Seo J.; Blundell C.; Kim H. C.; Lee E.; Lee S. H.; Moon A.; Moon W. K.; Huh D. A Microengineered Pathophysiological Model of Early-Stage Breast Cancer. Lab Chip 2015, 15, 3350 10.1039/C5LC00514K. 26158500 PMC4524879

[ref164] Ahn S. I.; Sei Y. J.; Park H. J.; Kim J.; Ryu Y.; Choi J. J.; Sung H. J.; MacDonald T. J.; Levey A. I.; Kim Y. T. Microengineered Human Blood-Brain Barrier Platform for Understanding Nanoparticle Transport Mechanisms. Nat. Commun. 2020, 10.1038/s41467-019-13896-7. PMC6954233 31924752

[ref165] Nair A. L.; Mesch L.; Schulz I.; Becker H.; Raible J.; Kiessling H.; Werner S.; Rothbauer U.; Schmees C.; Busche M.; Trennheuser S.; Fricker G.; Stelzle M. Parallelizable Microfluidic Platform to Model and Assess in Vitro Cellular Barriers: Technology and Application to Study the Interaction of 3D Tumor Spheroids with Cellular Barriers. Biosensors 2021, 11, 314 10.3390/bios11090314. 34562904 PMC8471981

[ref166] Steinmetz K. L.; Spack E. G. The Basics of Preclinical Drug Development for Neurodegenerative Disease Indications. BMC Neurol. 2009, 9, S2 10.1186/1471-2377-9-S1-S2. 19534731 PMC2697630

[ref167] Zurina I. M.; Gorkun A. A.; Dzhussoeva E. V.; Kolokoltsova T. D.; Markov D. D.; Kosheleva N. V.; Morozov S. G.; Saburina I. N. Human Melanocyte-Derived Spheroids: A Precise Test System for Drug Screening and a Multicellular Unit for Tissue Engineering. Front. Bioeng. Biotechnol. 2020, 10.3389/fbioe.2020.00540. PMC7287162 32582665

[ref168] Zhang Z.; Chen L.; Wang Y.; Zhang T.; Chen Y. C.; Yoon E. Label-Free Estimation of Therapeutic Efficacy on 3D Cancer Spheres Using Convolutional Neural Network Image Analysis. Anal. Chem. 2019, 91 (21), 14093–14100. 10.1021/acs.analchem.9b03896. 31601098 PMC13134704

[ref169] Fetah K. L.; DiPardo B. J.; Kongadzem E. M.; Tomlinson J. S.; Elzagheid A.; Elmusrati M.; Khademhosseini A.; Ashammakhi N. Cancer Modeling-on-a-Chip with Future Artificial Intelligence Integration. Small 2019, 15 (50), 1901985 10.1002/smll.201901985. PMC6929691 31724305

[ref170] Nashimoto Y.; Okada R.; Hanada S.; Arima Y.; Nishiyama K.; Miura T.; Yokokawa R. Vascularized Cancer on a Chip: The Effect of Perfusion on Growth and Drug Delivery of Tumor Spheroid. Biomaterials 2020, 229, 119547 10.1016/j.biomaterials.2019.119547. 31710953

[ref171] Ran R.; Wang H. F.; Hou F.; Liu Y.; Hui Y.; Petrovsky N.; Zhang F.; Zhao C. X. A Microfluidic Tumor-on-a-Chip for Assessing Multifunctional Liposomes’ Tumor Targeting and Anticancer Efficacy. Adv. Healthc. Mater. 2019, 8, 1900015 10.1002/adhm.201900015. 30868753

[ref172] Akay M.; Hite J.; Avci N. G.; Fan Y.; Akay Y.; Lu G.; Zhu J. J. Drug Screening of Human GBM Spheroids in Brain Cancer Chip. Sci. Rep. 2018, 10.1038/s41598-018-33641-2. PMC6194126 30337660

[ref173] Tavares R. S. N.; Phuong-Tao T.; Maschmeyer I.; Maria-Engler S. S.; Schäfer-Korting M.; Winter A.; Zoschke C.; Lauster R.; Marx U.; Gaspar L. R. Toxicity of Topically Applied Drugs beyond Skin Irritation: Static Skin Model vs. Two Organs-on-a-Chip. Int. J. Pharm. 2020, 589, 119788 10.1016/j.ijpharm.2020.119788. 32882369

[ref174] Cui X.; Hartanto Y.; Zhang H. Advances in Multicellular Spheroids Formation. Journal of the Royal Society Interface. 2017, 14, 20160877 10.1098/rsif.2016.0877. 28202590 PMC5332573

[ref175] Brown M. J.; Bahsoun S.; Morris M. A.; Akam E. C. Determining Conditions for Successful Culture of Multi-Cellular 3D Tumour Spheroids to Investigate the Effect of Mesenchymal Stem Cells on Breast Cancer Cell Invasiveness. Bioengineering 2019, 6, 101 10.3390/bioengineering6040101. 31683821 PMC6955867

[ref176] Mironov V.; Visconti R. P.; Kasyanov V.; Forgacs G.; Drake C. J.; Markwald R. R. Organ Printing: Tissue Spheroids as Building Blocks. Biomaterials 2009, 30, 2164 10.1016/j.biomaterials.2008.12.084. 19176247 PMC3773699

[ref177] Białkowska K.; Komorowski P.; Bryszewska M.; Miłowska K. Spheroids as a Type of Three-Dimensional Cell Cultures—Examples of Methods of Preparation and the Most Important Application. International Journal of Molecular Sciences. 2020, 21, 6225 10.3390/ijms21176225. 32872135 PMC7503223

[ref178] Nishikawa T.; Tanaka Y.; Nishikawa M.; Ogino Y.; Kusamori K.; Mizuno N.; Mizukami Y.; Shimizu K.; Konishi S.; Takahashi Y.; Takakura Y. Optimization of Albumin Secretion and Metabolic Activity of Cytochrome P450 1A1 of Human Hepatoblastoma HepG2 Cells in Multicellular Spheroids by Controlling Spheroid Size. Biol. Pharm. Bull. 2017, 40, 334 10.1248/bpb.b16-00833. 28250275

[ref179] Russo M.; Cejas C. M.; Pitingolo G. Advances in Microfluidic 3D Cell Culture for Preclinical Drug Development. Progress in Molecular Biology and Translational Science 2022, 187, 163 10.1016/bs.pmbts.2021.07.022. 35094774

[ref180] Marimuthu M.; Rousset N.; St-Georges-Robillard A.; Lateef M. A.; Ferland M.; Mes-Masson A. M.; Gervais T. Multi-Size Spheroid Formation Using Microfluidic Funnels. Lab Chip 2018, 18, 304 10.1039/C7LC00970D. 29211088

[ref181] Nath S.; Devi G. R. Three-Dimensional Culture Systems in Cancer Research: Focus on Tumor Spheroid Model. Pharmacology and Therapeutics. 2016, 163, 94 10.1016/j.pharmthera.2016.03.013. 27063403 PMC4961208

[ref182] Anada T.; Masuda T.; Honda Y.; Fukuda J.; Arai F.; Fukuda T.; Suzuki O. Three-Dimensional Cell Culture Device Utilizing Thin Membrane Deformation by Decompression. Sensors Actuators, B Chem. 2010, 147, 376 10.1016/j.snb.2010.01.065.

[ref183] Ziółkowska K.; Stelmachowska A.; Kwapiszewski R.; Chudy M.; Dybko A.; Brzózka Z. Long-Term Three-Dimensional Cell Culture and Anticancer Drug Activity Evaluation in a Microfluidic Chip. Biosens. Bioelectron. 2013, 40, 68 10.1016/j.bios.2012.06.017. 22770829

[ref184] Kamatar A.; Gunay G.; Acar H. Natural and Synthetic Biomaterials for Engineering Multicellular Tumor Spheroids. Polymers. 2020, 12, 2506 10.3390/polym12112506. 33126468 PMC7692845

[ref185] Caliari S. R.; Burdick J. A. A Practical Guide to Hydrogels for Cell Culture. Nature Methods. 2016, 13, 405 10.1038/nmeth.3839. 27123816 PMC5800304

[ref186] Ferreira L. P.; Gaspar V. M.; Mano J. F. Decellularized Extracellular Matrix for Bioengineering Physiomimetic 3D in Vitro Tumor Models. Trends in Biotechnology. 2020, 38, 1397 10.1016/j.tibtech.2020.04.006. 32416940

[ref187] Catoira M. C.; Fusaro L.; Di Francesco D.; Ramella M.; Boccafoschi F. Overview of Natural Hydrogels for Regenerative Medicine Applications. J. Mater. Sci. Mater. Med. 2019, 10.1007/s10856-019-6318-7. PMC6787111 31599365

[ref188] Reddy M. S. B.; Ponnamma D.; Choudhary R.; Sadasivuni K. K. A Comparative Review of Natural and Synthetic Biopolymer Composite Scaffolds. Polymers. 2021, 13, 1105 10.3390/polym13071105. 33808492 PMC8037451

[ref189] Bhatia S.; Bhatia S. Natural Polymers vs Synthetic Polymer. Natural Polymer Drug Delivery Systems 2016, 95 10.1007/978-3-319-41129-3_3.

[ref190] Zhang W.; Du A.; Liu S.; Lv M.; Chen S. Research Progress in Decellularized Extracellular Matrix-Derived Hydrogels. Regenerative Therapy. 2021, 18, 88 10.1016/j.reth.2021.04.002. 34095366 PMC8142036

[ref191] Garreta E.; Oria R.; Tarantino C.; Pla-Roca M.; Prado P.; Fernández-Avilés F.; Campistol J. M.; Samitier J.; Montserrat N. Tissue Engineering by Decellularization and 3D Bioprinting. Materials Today. 2017, 20, 166 10.1016/j.mattod.2016.12.005.

[ref192] Kwon J. S.; Oh J. H. Microfluidic Technology for Cell Manipulation. Applied Sciences (Switzerland). 2018, 8, 992 10.3390/app8060992.

[ref193] Azizipour N.; Avazpour R.; Sawan M.; Rosenzweig D. H.; Ajji A. Uniformity of Spheroids-on-a-Chip by Surface Treatment of PDMS Microfluidic Platforms. Sensors and Diagnostics 2022, 1, 750 10.1039/D2SD00004K.

[ref194] Ruppen J.; Wildhaber F. D.; Strub C.; Hall S. R. R.; Schmid R. A.; Geiser T.; Guenat O. T. Towards Personalized Medicine: Chemosensitivity Assays of Patient Lung Cancer Cell Spheroids in a Perfused Microfluidic Platform. Lab Chip 2015, 15, 3076 10.1039/C5LC00454C. 26088102

[ref195] Lee G.; Kim H.; Park J. Y.; Kim G.; Han J.; Chung S.; Yang J. H.; Jeon J. S.; Woo D. H.; Han C.; Kim S. K.; Park H. J.; Kim J. H. Generation of Uniform Liver Spheroids from Human Pluripotent Stem Cells for Imaging-Based Drug Toxicity Analysis. Biomaterials 2021, 269, 120529 10.1016/j.biomaterials.2020.120529. 33257114

[ref196] Wu L. Y.; Di Carlo D.; Lee L. P. Microfluidic Self-Assembly of Tumor Spheroids for Anticancer Drug Discovery. Biomed. Microdevices 2008, 10, 197 10.1007/s10544-007-9125-8. 17965938

[ref197] Hsiung L. C.; Chiang C. L.; Wang C. H.; Huang Y. H.; Kuo C. Te.; Cheng J. Y.; Lin C. H.; Wu V.; Chou H. Y.; Jong D. S.; Lee H.; Wo A. M. Dielectrophoresis-Based Cellular Microarray Chip for Anticancer Drug Screening in Perfusion Microenvironments. Lab Chip 2011, 11, 2333 10.1039/c1lc20147f. 21629948

[ref198] Elitas M.; Dhar N.; Schneider K.; Valero A.; Braschler T.; McKinney J. D.; Renaud P. Dielectrophoresis as a Single Cell Characterization Method for Bacteria. Biomed. Phys. Eng. Express 2017, 3, 015005 10.1088/2057-1976/3/1/015005.

[ref199] Caglayan Z.; Demircan Yalcın Y.; Kulah H. A Prominent Cell Manipulation Technique in Biomems: Dielectrophoresis. Micromachines 2020, 11, 990 10.3390/mi11110990. 33153069 PMC7693018

[ref200] Giduthuri A. T.; Theodossiou S. K.; Schiele N. R.; Srivastava S. K. Dielectrophoresis as a Tool for Electrophysiological Characterization of Stem Cells. Biophys. Rev. 2020, 1, 011304 10.1063/5.0025056. PMC10903368 38505626

[ref201] Delikoyun K.; Yaman S.; Yilmaz E.; Sarigil O.; Anil-Inevi M.; Telli K.; Yalcin-Ozuysal O.; Ozcivici E.; Tekin H. C. HologLev: A Hybrid Magnetic Levitation Platform Integrated with Lensless Holographic Microscopy for Density-Based Cell Analysis. ACS Sensors 2021, 6, 2191 10.1021/acssensors.0c02587. 34124887

[ref202] Duval K.; Grover H.; Han L. H.; Mou Y.; Pegoraro A. F.; Fredberg J.; Chen Z. Modeling Physiological Events in 2D vs. 3D Cell Culture. Physiology 2017, 32 (4), 266–277. 10.1152/physiol.00036.2016. 28615311 PMC5545611

[ref203] Kim J. B. Three-Dimensional Tissue Culture Models in Cancer Biology. Semin. Cancer Biol. 2005, 15, 365–377. 10.1016/j.semcancer.2005.05.002. 15975824

[ref204] Huang Y. L.; Segall J. E.; Wu M. Microfluidic Modeling of the Biophysical Microenvironment in Tumor Cell Invasion. Lab Chip 2017, 17 (19), 3221–3233. 10.1039/C7LC00623C. 28805874 PMC6007858

[ref205] Hong H. K.; Yun N. H.; Jeong Y.-L.; Park J.; Doh J.; Lee W. Y.; Cho Y. B. Establishment of Patient-Derived Organotypic Tumor Spheroid Models for Tumor Microenvironment Modeling. Cancer Med. 2021, 10, 5589–5598. 10.1002/cam4.4114. 34240815 PMC8366099

[ref206] Haddrick M.; Simpson P. B. Organ-on-a-Chip Technology: Turning Its Potential for Clinical Benefit into Reality. Drug Discovery Today. 2019, 24, 1217 10.1016/j.drudis.2019.03.011. 30880172

[ref207] Davies P. F. Flow-Mediated Endothelial Mechanotransduction. Physiological Reviews. 1995, 75, 519 10.1152/physrev.1995.75.3.519. 7624393 PMC3053532

[ref208] Chang S. F.; Chang C. A.; Lee D. Y.; Lee P. L.; Yeh Y. M.; Yeh C. R.; Cheng K.; Chien S.; Chiu J. J. Tumor Cell Cycle Arrest Induced by Shear Stress: Roles of Integrins and Smad. Proc. Natl. Acad. Sci. U. S. A. 2008, 105, 3927 10.1073/pnas.0712353105. 18310319 PMC2268796

[ref209] Theobald J.; Ghanem A.; Wallisch P.; Banaeiyan A. A.; Andrade-Navarro M. A.; Taškova K.; Haltmeier M.; Kurtz A.; Becker H.; Reuter S.; Mrowka R.; Cheng X.; Wölfl S. Liver-Kidney-on-Chip to Study Toxicity of Drug Metabolites. ACS Biomater. Sci. Eng. 2018, 4, 78 10.1021/acsbiomaterials.7b00417. 33418680

[ref210] Tian C.; Tu Q.; Liu W.; Wang J. Recent Advances in Microfluidic Technologies for Organ-on-a-Chip. TrAC - Trends in Analytical Chemistry. 2019, 117, 146 10.1016/j.trac.2019.06.005. PMC7434086 32831435

[ref211] Byun C. K.; Abi-Samra K.; Cho Y. K.; Takayama S. Pumps for Microfluidic Cell Culture. Electrophoresis. 2014, 35, 245 10.1002/elps.201300205. 23893649

[ref212] Watanabe S.; Inagaki S.; Kinouchi I.; Takai H.; Masuda Y.; Mizuno S. Hydrostatic Pressure/Perfusion Culture System Designed and Validated for Engineering Tissue. J. Biosci. Bioeng. 2005, 100, 105 10.1263/jbb.100.105. 16233859

[ref213] Choi Y. Y.; Kim J.; Lee S. H.; Kim D. S. Lab on a Chip-Based Hepatic Sinusoidal System Simulator for Optimal Primary Hepatocyte Culture. Biomed. Microdevices 2016, 10.1007/s10544-016-0079-6. 27334878

[ref214] McMillan K. S.; Boyd M.; Zagnoni M. Transitioning from Multi-Phase to Single-Phase Microfluidics for Long-Term Culture and Treatment of Multicellular Spheroids. Lab Chip 2016, 16, 3548 10.1039/C6LC00884D. 27477673

[ref215] Ota H.; Yamamoto R.; Deguchi K.; Tanaka Y.; Kazoe Y.; Sato Y.; Miki N. Three-Dimensional Spheroid-Forming Lab-on-a-Chip Using Micro-Rotational Flow. Sensors Actuators, B Chem. 2010, 147, 359 10.1016/j.snb.2009.11.061.

[ref216] Nge P. N.; Rogers C. I.; Woolley A. T. Advances in Microfluidic Materials, Functions, Integration, and Applications. Chem. Rev. 2013, 113, 2550 10.1021/cr300337x. 23410114 PMC3624029

[ref217] Raj M K.; Chakraborty S. PDMS Microfluidics: A Mini Review. J. Appl. Polym. Sci. 2020, 137 (27), 48958 10.1002/app.48958.

[ref218] Prabhakar P.; Sen R. K.; Dwivedi N.; Khan R.; Solanki P. R.; Srivastava A. K.; Dhand C. 3D-Printed Microfluidics and Potential Biomedical Applications. Front. Nanotechnol. 2021, 10.3389/fnano.2021.609355.

[ref219] Kecili S.; Tekin H. C. Adhesive Bonding Strategies to Fabricate High-Strength and Transparent 3D Printed Microfluidic Device. Biomicrofluidics 2020, 14, 024113 10.1063/5.0003302. 32341724 PMC7173975

[ref220] Wang N.; Liu R.; Asmare N.; Chu C. H.; Sarioglu A. F. Integrated Sensor Networks with Error Correction for Multiplexed Particle Tracking in Microfluidic Chips. Biosens. Bioelectron. 2021, 174, 112818 10.1016/j.bios.2020.112818. 33250334

[ref221] Grist S. M.; Nasseri S. S.; Laplatine L.; Schmok J. C.; Yao D.; Hua J.; Chrostowski L.; Cheung K. C. Long-Term Monitoring in a Microfluidic System to Study Tumour Spheroid Response to Chronic and Cycling Hypoxia. Sci. Rep. 2019, 9 (1), 1–13. 10.1038/s41598-019-54001-8. 31780697 PMC6883080

[ref222] Delikoyun K.; Cine E.; Anil-inevi M.; Sarigil O.; Ozcivici E.; Tekin H. C. 2 Deep Learning-Based Cellular Image Analysis for Intelligent Medical Diagnosis. In Artificial Intelligence for Data-Driven Medical Diagnosis; 2021; p 19 10.1515/9783110668322-002

[ref223] D Cell Culture Market Size, Share | 2022-2027 | MarketsandMarkets. https://www.marketsandmarkets.com/Market-Reports/3d-cell-culture-market-191072847.html (accessed 2022-10-20).

[ref224] Perfecta3D hanging drop plate size 96 wells, with lid and tray, polystyrene (untreated), sterile | Sigma-Aldrich. https://www.sigmaaldrich.com/TR/en/product/sigma/hdp1096?gclid=CjwKCAjwh4ObBhAzEiwAHzZYUwZoRTXwjJLFCxbwkLP-AVeEcH7fapP7ZXqPzoIJKfUuA9bL8FMpfBoCPCoQAvD_BwE&gclsrc=aw.ds (accessed 2022-11-02).

[ref225] Corning spheroid microplates. https://www.sigmaaldrich.com/TR/en/product/sigma/cls4520?gclid=CjwKCAjwh4ObBhAzEiwAHzZYU2PS2ODLsjj1WMuCT-Jpfs9W9lEsq2HOD6h2uO5TezXUAAQFIboPGhoCOJcQAvD_BwE&gclsrc=aw.ds (accessed 2022-11-02).

[ref226] MicroTissues 3D Petri Dish micro-mold spheroids size S, 16 × 16 array, fits 12 well plates 3D Cell Culture. https://www.sigmaaldrich.com/TR/en/product/sigma/z764000?gclid=CjwKCAjwh4ObBhAzEiwAHzZYUzgUqBmyjGlX85se9A11yIhgzUq0MxDMBiDwUmQP6yu04tIAtQlbkBoCs-UQAvD_BwE&gclsrc=aw.ds (accessed 2022-11-02).

[ref227] Utama R. H.; Atapattu L.; O’Mahony A. P.; Fife C. M.; Baek J.; Allard T.; O’Mahony K. J.; Ribeiro J. C. C.; Gaus K.; Kavallaris M.; Gooding J. J. A 3D Bioprinter Specifically Designed for the High-Throughput Production of Matrix-Embedded Multicellular Spheroids. iScience 2020, 23 (10), 101621 10.1016/j.isci.2020.101621. 33089109 PMC7567958

[ref228] Graham A. D.; Olof S. N.; Burke M. J.; Armstrong J. P. K.; Mikhailova E. A.; Nicholson J. G.; Box S. J.; Szele F. G.; Perriman A. W.; Bayley H. High-Resolution Patterned Cellular Constructs by Droplet-Based 3D Printing. Sci. Reports 2017 71 2017, 7 (1), 1–11. 10.1038/s41598-017-06358-x. PMC5539110 28765636

